# Nanoparticle display of neuraminidase elicits enhanced antibody responses and protection against influenza A virus challenge

**DOI:** 10.1038/s41541-024-00891-3

**Published:** 2024-05-31

**Authors:** M. N. Pascha, M. Ballegeer, M. C. Roelofs, L. Meuris, I. C. Albulescu, F. J. M. van Kuppeveld, D. L. Hurdiss, B. J. Bosch, T. Zeev-Ben-Mordehai, X. Saelens, C. A. M. de Haan

**Affiliations:** 1 Section of Virology, Division of Infectious Diseases & Immunology, Department of Biomolecular Health Sciences, Faculty of Veterinary Medicine, Utrecht University, Utrecht, The Netherlands; 2 VIB Center for Medical Biotechnology, VIB, 9052 Ghent, Belgium; 3 Department of Biochemistry and Microbiology, Ghent University, 9052 Ghent, Belgium; 4 Structural Biochemistry, Bijvoet Centre for Biomolecular Research, Department of Chemistry, Faculty of Science, Utrecht University, Utrecht, The Netherlands

**Keywords:** Protein vaccines, Influenza virus

## Abstract

Current Influenza virus vaccines primarily induce antibody responses against variable epitopes in hemagglutinin (HA), necessitating frequent updates. However, antibodies against neuraminidase (NA) can also confer protection against influenza, making NA an attractive target for the development of novel vaccines. In this study, we aimed to enhance the immunogenicity of recombinant NA antigens by presenting them multivalently on a nanoparticle carrier. Soluble tetrameric NA antigens of the N1 and N2 subtypes, confirmed to be correctly folded by cryo-electron microscopy structural analysis, were conjugated to Mi3 self-assembling protein nanoparticles using the SpyTag-SpyCatcher system. Immunization of mice with NA-Mi3 nanoparticles induced higher titers of NA-binding and -inhibiting antibodies and improved protection against a lethal challenge compared to unconjugated NA. Additionally, we explored the co-presentation of N1 and N2 antigens on the same Mi3 particles to create a mosaic vaccine candidate. These mosaic nanoparticles elicited antibody titers that were similar or superior to the homotypic nanoparticles and effectively protected against H1N1 and H3N2 challenge viruses. The NA-Mi3 nanoparticles represent a promising vaccine candidate that could complement HA-directed approaches for enhanced potency and broadened protection against influenza A virus.

## Introduction

H1N1 and H3N2 seasonal influenza A viruses (IAV) are responsible for the majority of the estimated 290,000–650,000 influenza-related deaths that occur annually worldwide^1^. Influenza vaccines are of critical importance in protecting high-risk groups from severe disease and mortality. Currently, available vaccines primarily induce antibodies targeting antigenically variable epitopes on hemagglutinin (HA)^2^. As a result, the protective efficacy of these vaccines is limited in large part by the antigenic match of the vaccine with the circulating IAV strains. One of the main challenges currently in the influenza field is, therefore, to develop vaccines that can offer broader protection and overcome the constraints imposed by antigenic variation.

IAV contains two glycoproteins with different functional roles; HA binds to sialoglycan receptors and mediates membrane fusion, whereas neuraminidase (NA) is the receptor-destroying enzyme that removes the terminal sialic acids. The sialidase activity of NA is essential for virion release from (decoy) receptors on host cells and for virion mobility through sialic acid-dense surroundings to reach functional entry receptors^3^. Antibodies that bind the HA head domain and thereby inhibit receptor binding are traditionally recognized as the main correlate of protection^4,5^. However, antibodies targeting NA also independently correlate with protection against disease and transmission of IAV^6–9^. The tetrameric NA protein consists of a globular head domain, containing the catalytic site, a narrow stalk, a transmembrane region, and a short cytoplasmic domain. NA-inhibiting (NAI) antibodies interfere with sialidase activity by binding directly to the catalytic site or by limiting the access of the sialic acids to the catalytic site through steric hindrance. Non-NAI antibodies may provide indirect protection through Fc-mediated effector functions^10–13^.

One strategy to induce NA-directed protective immunity is by vaccination with purified recombinant NA proteins^14–17^. The immunogenicity of such viral glycoprotein-based subunit vaccines can be improved considerably by the presentation of the antigen on a nanoparticle carrier. This has been demonstrated for various viral targets, including glycoproteins of coronavirus, respiratory syncytial virus (RSV), and Lassa virus^18–22^. Antigens conjugated to nanoparticle carriers more effectively activate the immune system because of increased size and antigen repetitiveness^23,24^. Nanoparticle vaccine platforms are also highly adaptable and allow for the conjugation of antigens of diverse symmetries^25^. Moreover, the presentation of multiple related, but antigenically different antigens, has been demonstrated to induce broadly cross-protective immune responses for IAV HA and coronavirus receptor binding domain^26–30^.

Here, we describe immunogenicity profiling and virus challenge studies in mice to evaluate NA nanoparticle vaccine candidates. We produced soluble recombinant tetrameric NA proteins stabilized with a tetrabrachion tetramerization domain, validated their quaternary structure by cryo-electron microscopy (cryo-EM) single-particle analysis, and conjugated these to Mi3 self-assembling protein nanoparticles using the SpyTag-SpyCatcher system^31,32^. Vaccination of mice with the resulting N1-Mi3 or N2-Mi3 nanoparticles induced high titers of NA-binding and inhibiting antibodies, and protected against an otherwise lethal challenge with H1N1 or H3N2 IAV, respectively. In addition, we co-presented the N1 and N2 antigens on Mi3 to create mosaic nanoparticles. These induced high antibody titers and provided potent protection against H1N1 and H3N2 challenge viruses. The humoral response and protection offered by the mosaic nanoparticles were as good or even superior to a mix of homotypic N1-Mi3 and N2-Mi3 nanoparticles.

## Results

### Recombinant tetrameric NA antigens

**Fig. 1:**
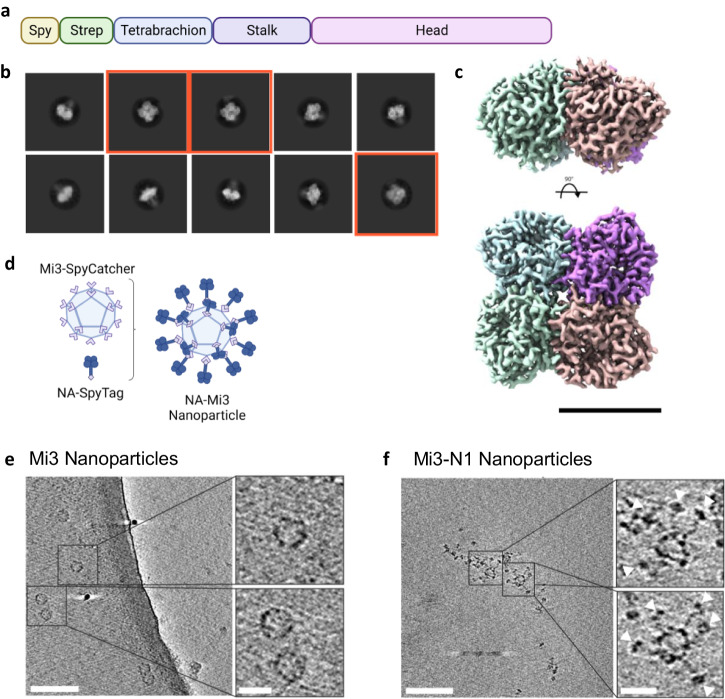
Recombinant NA antigens and coupling to Mi3 nanoparticles. **a** Schematic representation of the recombinant soluble NA protein constructs. NA proteins contain a SpyTag and Twin-Strep-tag, followed by a Tetrabrachion tetramerization domain fused to the NA stalk and head domain. **b** Cryo-EM 2D class averages of recombinant N1 NC13 stabilized with tetrabrachion, showing the closed conformation of the tetramer, top views marked in red. **c** Cryo-EM 3D reconstruction of recombinant N1 NC13 head domain, colored per individual protomer. The top panel shows a side-view and the bottom panel a view down the symmetry axis. Scalebar = 5 nm. **d** Schematic of NA-SpyTag conjugation to Mi3-SpyCatcher nanoparticles. Central slice through a tomogram with unconjugated Mi3 nanoparticles (**e**) and Mi3-N1 conjugated nanoparticles (**f**). Insets show enlarged areas in the left panel, and arrowheads in **f** indicate N1 tetramers. Scale bars in main micrographs = 100 nm, scale bars in inset panels = 25 nm.

We produced recombinant N1 NC13 (derived from A/North Carolina/07/2013 H1N1) and N2 HK68 (derived from A/Hong Kong/1968 (H3N2)) antigens in a soluble tetrameric form using a previously described construct design in which the transmembrane region is replaced with a tetrabrachion tetramerization domain^33^. In addition, the constructs contained a Strep-tag for affinity purification and a SpyTag for later conjugation to Mi3 nanoparticles (Fig. 1a). The antigens were produced in a mammalian (HEK293F) expression system, affinity purified, and evaluated for purity and size on SDS-PAGE (Supplementary Fig. 1a). The recombinant N1 and N2 proteins were highly enzymatically active in an assay based on the cleavage of the small molecule substrate MUNANA (Supplementary Fig. 1b).

The structural integrity of recombinant NA antigens is of critical importance for inducing an optimal immune response^34–36^. It was recently shown that recombinant soluble tetrameric NAs, particularly of the N1 subtype, may suffer from structural instability in the head domain, resulting in an “open” conformation not generally observed on virions^37,38^. Therefore, we performed cryo-EM to evaluate the quaternary structure of our constructs. 2D classification of the tetrabrachion-stabilized recombinant N1 NC13 produced class averages corresponding to different views of the NA tetramers, all of which were consistent with the closed conformation (Fig. 1b). The 3D reconstruction further confirmed this view (Fig. 1c, Supplementary Fig. 2a–c). Moreover, the atomic model built into the cryo-EM map shows high similarity to the previously published model based on X-ray crystallography (PDB: 3NSS, RMSD: 0.436 Å). N2 HK68 also displayed a closed conformation in negative stain EM (Supplementary Fig. 2d). Collectively, these observations indicate that our NA antigens adopt a closed conformation similar to the presumed conformation of NA on viral particles^37,38^.

### NA-Mi3 nanoparticle coupling

To construct the immunogens, we covalently attached NA proteins to Mi3 nanoparticles using the SpyTag/SpyCatcher system. The 60-meric Mi3 self-assembling protein nanoparticles with an N-terminal SpyCatcher domain were expressed in *E. coli*, affinity purified using the C-tag, and analyzed on SDS-PAGE (Supplementary Fig. 3a). Combining SpyCatcher-Mi3 nanoparticles with SpyTag-NA causes the spontaneous formation of an isopeptide bond that conjugates the NA to the nanoparticle (Fig. 1d). We evaluated the coupling efficiency at varying molar ratios and selected a ratio of 1:2 (NA:Mi3) for production of the immunogens as it resulted in the highest proportion of conjugated NA (Supplementary Fig. 3a). A portion of the NA remained unconjugated, presumably in part due to the symmetry mismatch between the Mi3 particles (3-fold symmetry) and NA (4-fold symmetry). Cryo-electron tomography (cryo-ET) analysis of undecorated nanoparticles (Fig. 1e) and nanoparticles decorated with N1 (Fig. 1f), demonstrate the conjugation of NA to the nanoparticles, confirming observations in negative stain projections (Supplementary Fig. 3b, c). The coupling of NA to Mi3 nanoparticles had no effect on its enzymatic activity as determined in the MUNANA assay (Supplementary Fig. 3d), suggesting that it did not impact the structural integrity of the NA proteins.

### NA-Mi3 nanoparticles induce higher serum antibody titers using less antigen

**Fig. 2:**
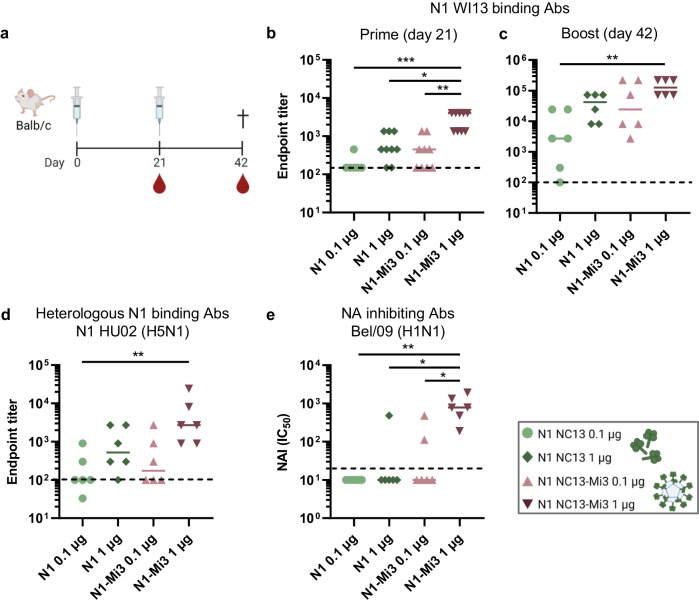
Immunization with N1-Mi3 nanoparticles induces high antibody titers. **a** Immunization scheme. BALB/c mice (*n* = 6) were immunized with N1 NC13 unconjugated and conjugated to Mi3 nanoparticles (N1-Mi3) in a prime-boost regimen at 0.1 or 1 µg per dose based on the total NA content. Serum samples were collected for serology analysis at day 21 (prime) and day 42 (boost) after primary immunization. **b**–**d** N1-binding antibodies in serum samples were quantified in ELISA and endpoint titers were determined as described in materials and methods. Endpoint titers against homologous N1 WI13 after prime (**b**) and boost (**c**) immunizations. **d** Endpoint titers against heterologous N1 HU02 (H5N1) after boost immunizations. **e** NA inhibiting antibodies in post boost sera were quantified in an ELLA assay with H1N1 Bel/09. IC_50_ values represent the reciprocal dilutions resulting in 50% inhibition of NA enzymatic activity. Bars represent the geometric means for each group (*n* = 6–9). The dotted lines represent the lowest serum dilution tested. Groups were compared with Kruskal-Wallis and Dunn’s post-hoc test using GraphPad Prism 9.3.1 (*P < 0.05; **P < 0.01; ***P < 0.001).

Vaccination of mice with the NA proteins formulated as unconjugated or nanoparticle-conjugated antigens was performed to test the hypothesis that nanoparticle presentation enhances the immunogenicity of NA. Mice were vaccinated with a low (0.1 µg NA) or high (1 µg NA) dose of the antigen and boosted three weeks later (Fig. 2a). All immunizations were performed by subcutaneous injections using Sigma Adjuvant System (SAS) as adjuvant. Mock-immunized mice received injections with PBS and SAS. Serology analysis was performed on sera collected three weeks after primary immunization (day 21 of the experiment, before administering the booster dose) and three weeks after the booster immunization (day 42) (Fig. 2a). NA-binding antibody titers were determined in enzyme-linked immunosorbent assays (ELISAs) using recombinant soluble NA proteins carrying a GCN4 tetramerization domain and a FLAG tag for purification, as to detect only NA-specific antibodies. NAI titers were determined in enzyme-linked lectin assays (ELLAs), measuring cleavage of the terminal sialic acids from the glycoprotein fetuin, using H1N1 Bel/09 or H3N2 X31 virus preparations.

Three weeks after the primary immunization, mice of all groups immunized with the N1 antigens developed a detectable antibody response against homologous N1 derived from A/Wisconsin/09/2013 (WI13). This N1 differs from the vaccine antigen N1 NC13 only at amino acid position 386^39^. Serum IgG titers were significantly higher in the mice that received the high dose of N1-Mi3 compared to all other groups (Fig. 2b). The booster immunization induced a significant increase in the NA-specific humoral responses (Fig. 2c and Supplementary Fig. 4a). High titers were observed for mice that received a high dose of N1 and either dose of N1-Mi3. Low dose N1-Mi3 immunized mice had comparable antibody titers to mice from the group that received a 10-fold higher dose of NA as an unconjugated antigen (Fig. 2c).

Mice in all N1-vaccinated groups also developed antibodies that cross-reacted with N1 derived from A/Hunan/795/2002, an avian H5N1 strain (HU02; 87.5% shared sequence identity with N1 NC13) (Fig. 2d). Reactivity against this heterologous N1 was lower overall, but followed similar trends to those observed for homologous N1. The highest cross-reactive titers were detected in mice immunized with a high dose of N1-Mi3. The induction of antibodies capable of inhibiting sialidase activity was tested in an ELLA assay against a 2009 pandemic H1N1 virus (Bel/09). The NA protein of this virus shares 98% sequence identity with the N1 NC13 protein that was used for the immunizations. Consistent with the total NA-binding antibody titers, NAI titers were highest in sera from mice immunized with high dose N1-Mi3 (Fig. 2e). Detectable NAI antibodies were only found in 3 out of 18 mice from the other groups.

**Fig. 3:**
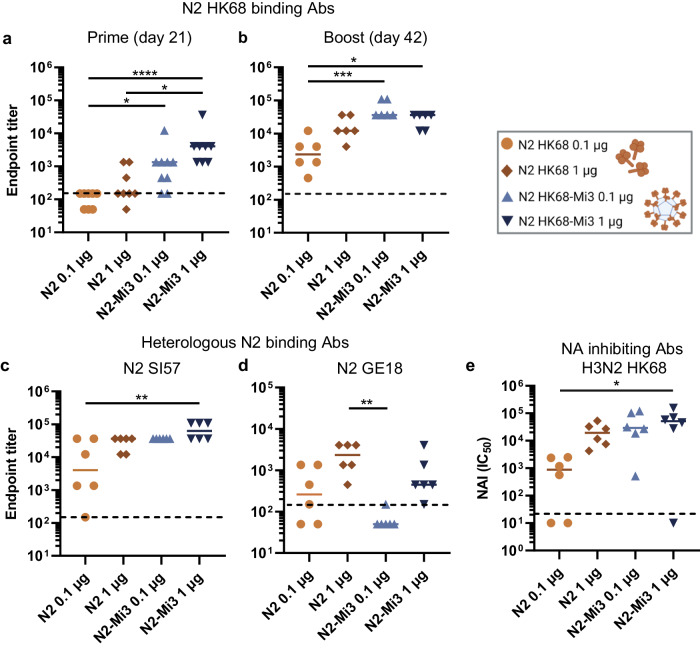
Immunization with N2-Mi3 nanoparticles induces high antibody titers. BALB/c mice were immunized with N2 HK68 unconjugated and coupled to Mi3 (N2-Mi3) in a prime-boost regimen at 0.1 or 1 µg per dose based on the total NA content. Serum samples were collected for serology analysis at day 21 (prime) and day 42 (boost) after primary immunization. N2-binding antibodies in serum samples were quantified in ELISA and endpoint titers were determined as described in materials and methods. (a and b) Endpoint titers against homologous N2 HK68 after prime (**a**) and boost (**b**) immunizations. Endpoint titers against heterologous N2 SI57 (**c**) and N2 GE18 (**d**) after boost immunizations. **e** NA-inhibiting antibodies in post-boost sera were quantified in an ELLA assay with H3N2 HK68. IC_50_ values represent the reciprocal dilutions resulting in 50% inhibition of NA enzymatic activity. Bars represent the geometric means for each group (*n* = 6–9). The dotted lines represent the lowest serum dilution tested. Groups were compared with Kruskal-Wallis and Dunn’s post-hoc test using GraphPad Prism 9.3.1 (*P < 0.05; **P < 0.01; ***P < 0.001; ****P < 0.0001).

A similar prime-boost immunization experiment was performed using N2 antigens. Most of the mice immunized with the N2 antigens developed measurable antibody titers after primary immunization. The N2-Mi3 vaccinated mice had significantly higher antibody titers against the homologous N2 antigen (N2 HK68) than the mice that received unconjugated N2 antigens at an equivalent dosage (Fig. 3a). The highest serum IgG titers were again observed for the mice that received the high dose of the Mi3-coupled NAs. Antibody titers were further increased after the booster vaccination (Fig. 3b and Supplementary Fig. 4b). Mice immunized with low or high doses of N2-Mi3 had significantly higher levels of antibodies than the low dose unconjugated N2 immunized group (Fig. 3b).

We next evaluated cross-reactivity of the immune sera to heterologous N2 NAs. High titers of cross-reactive antibodies against NA of a 1957 H2N2 strain (N2 SI57; 95% shared sequence identity with N2 HK68) were detected in all test groups, similar to the reactivity measured against the homologous NA (Fig. 3b, c). Reactivity against the antigenically drifted NA of 2018 H3N2 virus (N2 GE18; 84.1% shared sequence identity with N2 HK68) was lower in all groups. Interestingly, titers against this antigen appeared lower in the N2-Mi3 vaccinated mice than in the unconjugated N2 vaccinated mice. Only 1 out of 6 mice that received the low dose N2-Mi3 developed measurable antibodies against GE18 (Fig. 3d). These data suggest that the presentation of N2 on nanoparticles resulted in targeting of different, more antigenically variable epitopes, compared to unconjugated N2.

We analyzed the induction of NAI antibodies in mice immunized with the N2 antigens in an ELLA assay using the H3N2 X31 virus, which carries an NA homologous to the N2 HK68 that was used for the immunizations. High titers of NAI antibodies were elicited by the high dose unconjugated N2 immunization and both dosages of N2-Mi3 (Fig. 3e). These results correlate with the ELISA antibody titers against N2 HK68 (Fig. 3b), although two N2-Mi3 vaccinated animals responded with much lower or undetectable NAI titers (Fig. 3e). Notably, the NAI titers against H3N2 HK68 in these N2-vaccinated mice were higher than the titers against H1N1 Bel/09 in the N1-vaccinated mice. This difference may be explained in part by the lower NA activity of the H1N1 virus preparations compared to H3N2 (Supplementary Fig. 5). Because of the lower NA activity, more virus was used in the ELLA-based NAI assay for Bel/09, likely resulting in a higher virion-to-antibody ratio.

Taken together, we found that NA presented on Mi3 nanoparticles induced higher titers of total NA-binding serum antibodies against homologous NAs, compared to unconjugated NA, particularly after the prime immunization. A tenfold lower dose of NA-Mi3-induced antibody titers equal to or exceeding the titers induced with unconjugated NA. N1-Mi3 vaccination additionally induced higher NAI titers than unconjugated N1, whereas differences were not significant for N2.

### NA-Mi3 nanoparticles induce improved protection against homologous influenza A virus challenge

**Fig. 4:**
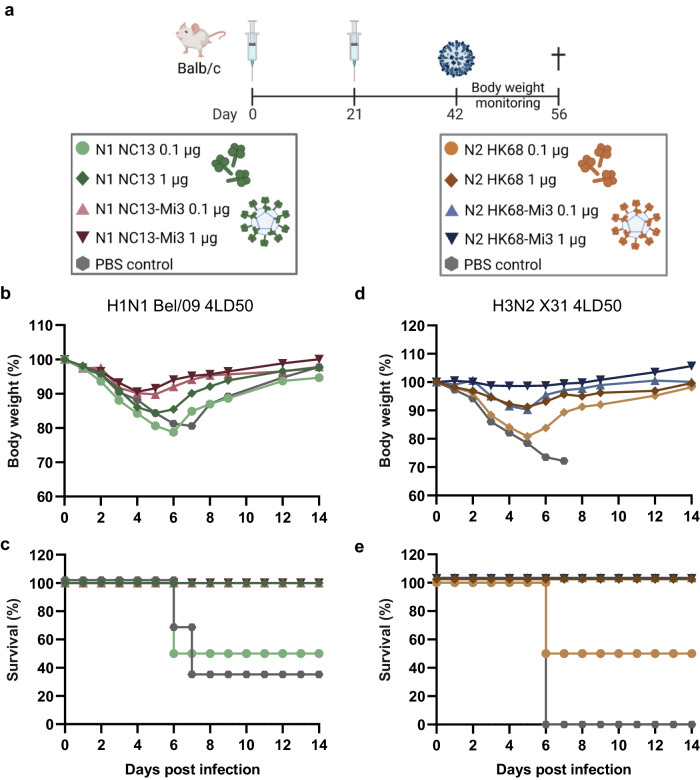
NA nanoparticle vaccination protects mice from homologous virus challenge. **a** Immunization and challenge schedule. BALB/c mice (*n* = 6 per group) were immunized with unconjugated NA or NA coupled to Mi3 in a prime-boost regimen at 0.1 or 1 µg per dose based on the total NA content. On day 42, N1- and N2-vaccinated mice were challenged with 4LD50 H1N1 Bel/09 or H3N2 X31, respectively. Mice were monitored for weight loss and survival for two weeks post-challenge. Mean body weights of mice immunized with N1 and N1-Mi3 after challenge with H1N1 (**b**) and of mice immunized with N2 or N2-Mi3 after challenge with H3N2 (**d**). Survival of mice immunized with N1 or N1-Mi3 after challenge with H1N1 (**c**) and of mice immunized with N2 or N2-Mi3 after challenge with H3N2 (**e**).

Next, we tested the protective efficacy of N1 and N2 formulated as unconjugated or nanoparticle-based vaccines against lethal homologous IAV challenge in a mouse model (Fig. 4). Three weeks after the second vaccine dose, mice (*n* = 6/group) were challenged with four times the lethal dose 50 (LD_50_) of H1N1 Bel/09 or H3N2 X31 (Fig. 4a). Changes in body weight were monitored for the next two weeks post-infection. N1-Mi3 vaccination protected mice against H1N1 Bel/09 challenge. Mice vaccinated with the nanoparticles were better protected against weight loss in the initial four days post-challenge (*p* = 0.006) and recovered faster (*p* = 0.007) compared to mice vaccinated with unconjugated N1 (Fig. 4b and Table 1). No significant differences in weight loss rates were detected between mice vaccinated with low or high doses of the vaccine preparations. All mice that received the N1-Mi3 vaccine, both at high and low doses, survived the challenge, as well as all mice that received the high dose of unconjugated N1. Only 3 out of 6 mice immunized with a low dose of unconjugated N1 and 2 out of 6 mock-immunized mice survived the challenge (Fig. 4c).

**Table 1:** Statistical analysis of body weight changes in mice immunized with unconjugated and nanoparticle-conjugated N1 NC13 after challenge with H1N1 Bel/09

H1N1 Bel/09	Adj. *p*-value^1^
Weight loss phase (day 0–4)	
1) N1 NC13-Mi3 vs. N1 NC13^2^	0.006
2) 1 vs. 0.1 µg N1 NC13	1
3) 1 vs. 0.1 µg N1 NC13-Mi3	1
Recovery phase (day 4–6)	
1) N1 NC13-Mi3 vs. N1 NC13^2^	0.007
2) 1 vs. 0.1 µg N1 NC13	0.22
3) 1 vs. 0.1 µg N1 NC13-Mi3	1

^1^Adjusted p-values for differences between groups shown in Fig. 4b in two phases of infection based on the piecewise linear mixed model output described in Supplementary Note 1.

^2^Comparison between mice immunized with N1-Mi3 and N1, combining the data from mice immunized with 0.1 and 1 µg of the antigens.

Similarly, N2-Mi3 vaccination provided better protection against H3N2 challenge than unconjugated N2, resulting in reduced weight loss in the initial phase of infection (*p* < 0.001) (Fig. 4d and Table 2). Mice immunized with the high dose of the nanoparticle vaccine demonstrated no significant change in body weight. Low-dose N2-Mi3 immunized mice experienced greater weight loss (*p* < 0.001), at a rate similar to the mice immunized with the high-dose unconjugated N2. All mice in these groups survived the challenge (Fig. 4e). Low-dose unconjugated N2 was less protective, as these mice experienced even greater weight loss (*p* < 0.001) and only 3 out of 6 mice of this group survived the challenge. For comparison, none of the mock-immunized mice survived the challenge.

**Table 2:** Statistical analysis of body weight changes in mice immunized with unconjugated and nanoparticle-conjugated N2 HK68 after challenge with H3N2 X31

H3N2 X31	Adj. p-value^1^
Weight loss phase (days 0–4)	
1) N2 HK68-Mi3 vs. N2 HK68^2^	<0.001
2) 1 vs. 0.1 µg N2 HK68	<0.001
3) 1 vs. 0.1 µg N2 HK68-Mi3	<0.001
Recovery phase (day 4–6)	
1) N2 HK68-Mi3 vs. N2 HK68^2^	ND^3^
2) 1 vs. 0.1 µg N2 HK68	0.552
3) 1 vs. 0.1 µg N2 HK68-Mi3	ND

^1^Adjusted p-values for differences between groups shown in Fig. 4d in two phases of infection based on the piecewise linear mixed model output described in Supplementary Note 1.

^2^Comparison between mice immunized with N2-Mi3 and N2, combining the data from mice immunized with 0.1 and 1 µg of the antigens.

^3^ND, not determined

In conclusion, vaccination with NA presented on Mi3 nanoparticles induced more potent protection against an otherwise lethal viral challenge than unconjugated NA. In agreement with the serology data, the protection provided by the low dose NA-Mi3 was similar (N2) or superior (N1) compared to a tenfold higher dose of unconjugated NA.

### Mosaic N1-N2-Mi3 nanoparticles induce high antibody titers against both NAs

After having established that the NA nanoparticle-based vaccines induce superior antibody titers and protection against IAV challenge, we next aimed to design a vaccine that could offer protection against H1N1 and H3N2 IAVs. N1 and N2 were combined in one vaccine formulation in two ways: (1) mixing the homotypic N1-Mi3 and N2-Mi3 particles (N1/N2-Mi3 mix vaccine) and (2) coupling N1 and N2 onto the same Mi3 particles (N1-N2-Mi3 mosaic vaccine). The effects of combining NAs of different subtypes on the antibody response and protection from IAV challenge were determined and compared to vaccination with homotypic N1-Mi3 or N2-Mi3 particles. The total NA content of the vaccines was kept constant at 0.2 µg per dose. Mice (*n* = 6 per group) were immunized following the same prime-boost regimen as described earlier (Fig. 2a). This experiment was performed twice and the combined results of the two experiments are shown.

**Fig. 5:**
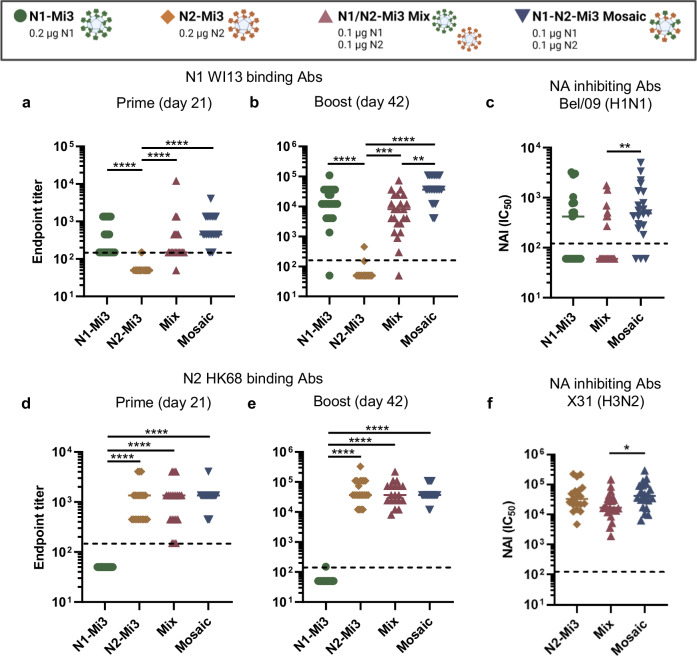
Nanoparticle vaccines combining N1 and N2 induce high antibody titers against both NAs. BALB/c mice (*n* = 12 in two independent experiments of 6 mice per group) were immunized with NA-Mi3 nanoparticles at 0.2 µg of NA per dose. Serum samples were collected for serology analysis on day 21 (prime) and day 42 (boost) after primary immunization. (**a**, **b**, **d**, and **e**) NA-binding antibodies in serum samples were quantified in ELISA. Endpoint titers against N1 WI13 after prime (**a**) and boost (**b**) immunizations. **c** NAI titers of H1N1 Bel/09 in post-boost sera were quantified in ELLA assay. IC_50_ values represent the reciprocal dilutions resulting in 50% inhibition of NA enzymatic activity. Endpoint titers against N2 HK68 after prime (**d**) and boost (**e**) immunizations. **f** NAI titers of H3N2 HK68 in post boost sera were quantified in ELLA assay. Horizontal bars represent the geometric means for each group. The dotted lines represent the lowest serum dilution tested. Groups were compared with Kruskal-Wallis and Dunn’s post-hoc test using GraphPad Prism 9.3.1 (*P < 0.05; **P < 0.01; ***P < 0.001; ****P < 0.0001).

All N1-containing vaccine preparations induced high antibody titers against the homologous N1 WI13, while no reactivity against N1 was observed after vaccination with the N2-Mi3 homotypic nanoparticles (Fig. 5a–c). An inversed pattern was observed for the N2-containing vaccines (Fig. 5d–f). Closer inspection of the serology data against N1 shows that the mixed particles induced NA-binding antibodies to similar titers as homotypic N1-Mi3, while the mosaic particles induced higher titers than the mixed particles after the boost immunizations (Fig. 5b). Moreover, the mosaic particles induced higher levels of NAI antibodies against NA of H1N1 than the mixed particles. No significant difference in NAI titers was observed between N1-N2-Mi3 mosaic particles and homotypic N1-Mi3 (Fig. 5c), consistent with the total NA binding antibody titers. Although the mosaic nanoparticles elicited a superior antibody response against N1 compared to the mixed nanoparticles, a similar effect was not observed for the overall reactivity against N2. The anti-N2 titers did not significantly differ between the groups immunized with the N2-Mi3, mixed, or mosaic nanoparticles at either time point in the experiment (Fig. 5d, e). However, the mosaic nanoparticles appeared to induce slightly higher NAI titers against H3N2 than the mixed nanoparticles (Fig. 5f).

To determine whether combining N1 and N2 antigens in one vaccine formulation can alter the breadth of the immune response, serum reactivity was tested against heterologous N1 and N2 NAs in ELISA. Results obtained with pooled serum samples from mice immunized with the different nanoparticle preparations were similar against homologous NAs (Supplementary Fig. 6a, c) as those obtained with the individual serum samples (Fig. 5b, e). No differences in cross-reactive responses were detected between the different preparations against any of the heterologous recombinant NAs that were tested (Supplementary Fig. 6). Furthermore, pooled serum samples from mice immunized with the different nanoparticle preparations were all unable to inhibit cleavage of MUNANA by NA (Supplementary Fig. 7), indicating the absence of antibodies targeting the conserved catalytic site.

Collectively, the serology data suggest superior performance of the N1-N2-Mi3 mosaic nanoparticles compared to the N1/N2-Mi3 mix, but not the homotypic NA-Mi3 vaccines. However, combining different NA subtypes in one vaccine did not measurably improve the breadth of the antibody response against heterologous N1 and N2 NAs.

In addition to characterizing the NA-targeting antibody response, we also evaluated the induction of antibodies directed against the Mi3 scaffold. High titers of anti-Mi3 antibodies were detected in all mice immunized with the NA-Mi3 nanoparticles. (Supplementary Fig. 8). These titers were comparable to the NA-specific titers, suggesting that both components of the NA-Mi3 nanoparticles are equally immunogenic when covalently linked. Interestingly, a substantially lower anti-scaffold response was detected in mice immunized with unconjugated Mi3 nanoparticles. Thus, the conjugation of NA results in an increased antibody response against the Mi3 scaffold.

### Mosaic nanoparticles protect against lethal H1N1 and H3N2 challenge

Finally, we assessed if vaccination with the nanoparticle-based vaccines that combine N1 and N2 antigens in the mixed or the mosaic formulation can protect against H1N1 and H3N2 virus challenge. Mice were challenged with H1N1 or H3N2 three weeks after the booster immunization and evaluated for weight loss and survival. Single homotypic N1-Mi3 and N2-Mi3 nanoparticles, and unconjugated Mi3 nanoparticles were included as controls.

**Fig. 6:**
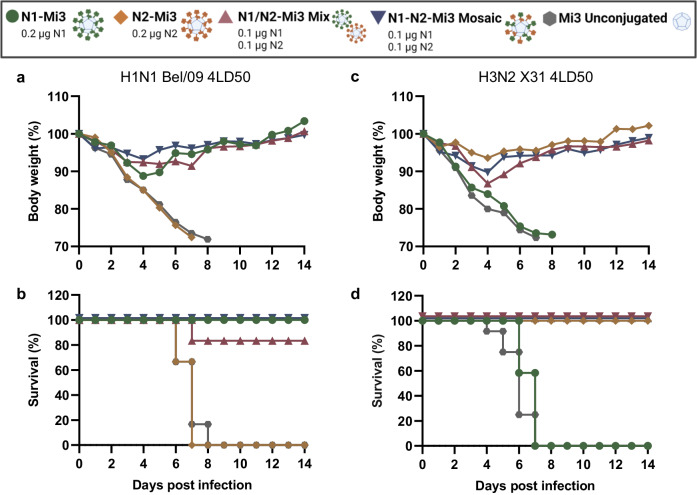
NA nanoparticle vaccination protects mice from H1N1 and H3N2 challenge. BALB/c mice (*n* = 12 in two independent experiments of 6 mice per group) were immunized with NA-Mi3 nanoparticles. Three weeks post-boost, N1- and N2-vaccinated mice were challenged with 4LD50 H1N1 Bel/09 or H3N2 X31, respectively. Mice were monitored for weight loss and survival for two weeks post-challenge. Mean body weights of mice immunized with NA-Mi3 after challenge with H1N1 (**a**) or H3N2 (**b**). Survival of mice immunized with NA-Mi3 after challenge with H1N1 (**c**) or H3N2 (**d**).

Immunization with single homotypic NA-Mi3 vaccine preparations protected mice against weight loss and lethality when challenged with the homologous, but not the heterosubtypic virus (Fig. 6), in accordance with the serology analyses (Fig. 5). Mice immunized with the mosaic particles experienced only minimal weight loss after H1N1 challenge, which resolved within a week. N1-Mi3 vaccinated mice lost weight more rapidly in the first four days post-challenge with H1N1 than mosaic vaccinated mice (*p* = 0.001), but also made a full recovery. In the N1/N2-Mi3 mix-immunized group, most animals suffered from transient weight loss (Fig. 6a), but 2 out of 12 mice continued to deteriorate and did not survive the challenge (Fig. 6b). The rate at which the N1/N2-Mi3 mix-immunized mice lost weight in the initial phase did not differ significantly from the mice immunized with N1-Mi3 (*p* = 0.145) or with the mosaic nanoparticles (*p* = 0.353) (Table 3). The N2-Mi3 group and a control group immunized with unconjugated Mi3 particles both experienced rapidly progressing weight loss and reached ethical endpoint criteria by day 6–8.

**Table 3:** Statistical analysis of body weight changes in mice immunized with different nanoparticles preparations after challenge with H1N1 Bel/09 or H3N2 X31

Weight loss phase (days 0–4)	Adj. *p*-value^a1^
H1N1 Bel/09	
1) N1-Mi3 vs. N1-N2-Mi3 Mosaic	0.001
2) N1-Mi3 vs. N1/N2 Mix	0.145
3) N1/N2-Mi3 Mix vs. N1-N2-Mi3 Mosaic	0.353
H3N2 X31	
1) N2-Mi3 vs. N1-N2-Mi3 Mosaic	0.992
2) N2-Mi3 vs. N1/N2 Mix	0.002
3) N1/N2-Mi3 Mix vs. N1-N2-Mi3 Mosaic	0.472

^1^Adjusted p-values for differences between groups in the initial weight loss phase of infection based on the piecewise linear mixed model output described in Supplementary Note 1.

Similarly, immunizations with the N2-containing nanoparticle formulations, but not with N1-Mi3 or unconjugated particles, protected mice against H3N2 challenge. All mice that received vaccines including N2 antigens experienced transient weight loss (Fig. 6c) and made a full recovery (Fig. 6d). In contrast, mice immunized with N1-Mi3 or empty Mi3 particles all died or reached ethical endpoint criteria by day 7 (Fig. 6d). The N2-Mi3 homotypic nanoparticles appeared to protect best against weight loss following H3N2 challenge. Weight loss rate in the four days post-challenge was lower in the mice immunized with N2-Mi3 compared to the mix immunized group (*p* = 0.002). No significant difference was observed between N2-Mi3 and the mosaic particles (*p* = 0.992) or mix and mosaic particles (*p* = 0.472) (Table 3).

We conclude that the N1-N2-Mi3 mosaic nanoparticles fully protect mice against both H1N1 and H3N2 challenge. A protective immune response was also induced by a mix of homotypic nanoparticles (N1/N2-Mi3 mix). However, in agreement with the serological analysis (Fig. 5), the protective efficacy of the mosaic nanoparticles in the H1N1 challenge appeared more robust compared to that of the mixed nanoparticle vaccine.

## Discussion

NA-targeting antibodies can protect against IAV infection, disease, and transmission^6,14,17,40,41^, but are poorly induced by currently licensed influenza vaccines^2^. Since the antigenic drift of NA is discordant with that of HA^42^, enhancing the antibody response against NA could potentially improve protection against drifted strains. Here, we show that multivalent presentation of tetrameric NA antigens on a self-assembling protein nanoparticle enhances their immunogenicity and results in improved protection from lethal IAV challenge. Additionally, we demonstrate that combining N1 and N2 NA antigens on mosaic nanoparticles is an effective strategy to induce protective immunity against both human H1N1 and H3N2 IAV subtypes.

The structural integrity of NA antigens is crucial for the induction of a qualitative immune response. It was recently shown that in some recombinant soluble NAs, in particular those of the N1 subtype, the head domain exhibits a more open or disordered conformation. Extensive mutational stabilization was required to change these into tightly packed, symmetric tetramers that resemble the native NA as it is presumably present on viral particles^37,38^. In contrast, our NA antigens adopt the closed conformation without the need for stabilizing mutations. This difference may be attributed to the different tetramerization domains that were used. The recombinant NAs that displayed a more open head assembly were fused to a tetramerization domain derived from human vasodilator-stimulated phosphoprotein (VASP), which has a coiled-coil structure with right-handed rotation^37^. The tetrabrachion coiled-coil that was used in our study has a more parallel structure and is suggested to provide higher stability to recombinant NAs^36,43^ and to result in NAs with higher enzymatic activity^33^. These findings suggest that using a better-suited tetramerization domain may obviate the need for mutagenesis to produce recombinant NAs that adopt the closed conformation. Nevertheless, the stabilizing mutations reported by Ellis and colleagues may further enhance the protein stability.

Our recombinant NA antigens are immunogenic and can induce a protective immune response against IAV challenge in mice. Consistent with previous studies on NA as well as other viral glycoproteins^18–22,25^, we found that the immunogenicity of NA can be enhanced by presenting it on nanoparticles. Nanoparticle-based immunogens elicit a stronger humoral immune response, characterized by higher antibody titers and possibly with higher affinity^22^. Antigen conjugation to a nanoparticle carrier results in immunogens that mimic the size and repetitiveness of the antigens on the virus particle. These characteristics promote the induction of a robust immune response on various levels, including increased innate immune activation, improved drainage to the lymph nodes, and cross-linking of B cell receptors leading to stronger B cell activation^23,24^. Although we cannot exclude that the Mi3 nanoparticles themselves have adjuvanting properties, this was observed not to be the case for similar Lumazine synthase nanoparticles in combination with SpyTag/SpyCatcher technology^22^. Our NA-Mi3 nanoparticles achieved a tenfold dose-sparing effect compared to unconjugated NA. Thus, nanoparticle presentation of NA could be an effective strategy for antigen sparing in addition to the use of adjuvants. This could be particularly valuable in a pandemic context.

In addition to the on-target NA-directed antibody response, NA-Mi3 immunization induced high levels of antibodies targeting the Mi3 nanoparticles. Notably, anti-Mi3 antibody titers were five- to tenfold higher in mice immunized with NA-Mi3 compared to those immunized with unconjugated Mi3 nanoparticles. This heightened anti-carrier response is not consistently observed for other nanoparticle-based immunogens. For instance, the conjugation of RSV F glycoproteins to different types of nanoparticles reduced the anti-scaffold response, indicating antigenic competition favoring the glycoproteins^20,44^. The balance between antigen- and scaffold-specific immunity against nanoparticle-based immunogens arises from intricate interactions within the immune system, providing multiple possible explanations for our observations. NA can engage CD4^+^ T cell help^45,46^, potentially amplifying the B cell response against both components of the immunogens. Additionally, NA’s sialidase activity may influence the reactivity against the Mi3 scaffold. By removing sialic acids from the cell surface, NA can contribute to the activation of various immune cell subsets, including B and T cells^47,48^. Thus, the presence of exogenous NA in germinal centers might foster an environment conductive to immune activation against the nanoparticle scaffold. It would therefore be insightful to compare the immunogenicity of enzymatically active and non-active NA preparations. The high reactivity observed against the Mi3 particles may raise concerns that anti-scaffold antibody responses could undermine the antigen-specific response, a phenomenon observed with the HIV-1 envelope glycoprotein^44,49^. Yet, such negative effects were not observed for other, more immunogenic viral glycoproteins^20,44,50^. CD4^+^ T cells targeting the scaffold might even positively contribute to the antigen-specific antibody response, as proposed for HA-ferritin nanoparticles^51^. Moreover, pre-existing antibodies directed against the tetrabrachion domain have been documented to enhance the immune response against tetrabrachion-stabilized NA, possibly by promoting antigen processing via immune complex formation^43^. Further research is needed to investigate if and how (pre-existing) anti-Mi3 responses influence the induction of NA-targeting antibodies.

Nanoparticle presentation not only amplifies the magnitude of the antibody response, but also modifies epitope targeting. Antigens displayed on a nanoparticle maintain a rigid outward orientation, mirroring their natural positioning on the viral particle. The orientation of an antigen on a nanoparticle is thought to affect its immunogenicity profile by making outward-directed epitopes more accessible for binding by B cell receptors^52^. This effect was demonstrated and taken advantage of with HA coupled to nanoparticles in an inverted orientation. Having the HA stalk directed outward resulted in the induction of five- to tenfold higher antibody titers against this more conserved domain^52^. In the current study, we observed that antibodies cross-reactive with N2 GE18 were induced to lower levels by N2-Mi3 nanoparticles compared to unconjugated N2 (Fig. 3d). It seems that epitopes conserved between the N2 of HK68 and GE18 were less efficiently targeted on the nanoparticle-conjugated N2, possibly as a result of reduced accessibility. In line with this hypothesis, cross-reactive antibodies targeting the underside of NA head domain have recently been described^53,54^. Given the outward-facing orientation of NA on the nanoparticles, the antibody response against NA-Mi3 is presumably directed mainly towards epitopes located at the top of the globular head, which might be more antigenically variable. Of note, despite observing diminished reactivity against N2 GE18, cross-reactivity with N2 from 1957 H2N2 was evident after immunization with N2-Mi3. Similarly, N1-Mi3 induced a cross-reactive response with N1 of an avian H5N1 virus. The increased reactivity against these heterologous NA proteins upon immunization with the NA-Mi3 preparations compared to the unconjugated NAs appeared proportional to the increases observed in total antibody titers against the homologous NAs, indicating that it reflects differences in the quantity rather than the breadth of the induced antibodies. These results imply that the immune response to NA-Mi3 may possess the breadth needed to protect against ‘recently’-drifted strains, although further work is needed to establish the breadth of the antibody response induced by homotypic NA-Mi3.

Furthermore, we demonstrate that mosaic nanoparticles induce a more balanced antibody response against both N1 and N2 immunogens than the mix of homotypic nanoparticles, in which the N1-specific response was somewhat lower. This disparity might stem from antigenic competition between the two NA proteins, which appears to affect these preparations differently. However, in prior immunizations with unconjugated N1 and N2, no competition between the antigens was reported^55^. Combining the antigens on one particle might also locally enhance the recruitment of CD4^+^ T cell help, resulting in enhanced reactivity compared to the mixed vaccine formulation. While the exact mechanism underlying the superiority of the mosaic NA nanoparticles remains unclear at present, these encouraging results could motivate further refinement, following the example of recently reported broadly reactive mosaic vaccines against influenza HA or coronavirus receptor binding domain^26–30^. These nanoparticles incorporate antigens of as many as eight different variants which ensures minimal clustering of identical antigens adjacent to each other. This characteristic is believed to provide an avidity advantage for B cells interacting with conserved epitopes, thereby boosting the induction of cross-reactive antibodies^26,28^. Further improvements of the mosaic NA nanoparticles, such as the inclusion of multiple NA subtypes, may enhance cross-reactivity by promoting the generation of antibodies targeting conserved epitopes, such as the catalytic site. Notably, certain antibodies targeting the catalytic site have demonstrated exceptional breadth^56,57^. The induction of such antibodies by vaccination would be expected to result in heterosubtypic immunity, a phenomenon rarely observed with experimental NA-based vaccines^13^. The current display of only two NA subtypes on the mosaic nanoparticles does not appear sufficient, however, to focus the immune response to the catalytic site and to result in the induction of broader-reactive antibodies compared to a mix of homotypic nanoparticles.

In conclusion, our study confirms the potency and adaptability of Mi3-SpyCatcher nanoparticles as a vaccine platform. We have demonstrated their capability to elicit a robust humoral immune response against NA and harnessed their versatility to induce a broadly reactive immune response against two highly divergent subtypes. Further refinement of the design holds the potential to enhance the breadth of the anti-NA response. Nonetheless, for achieving a robust and long-lasting immune response against multiple influenza virus subtypes and drifted strains, we anticipate that a multicomponent vaccine will be required. NA nanoparticles could complement HA-directed strategies, including inactivated virus vaccines or the more novel HA stem-based immunogens, to enhance and broaden their protective efficacy.

## Methods

### NA gene construct design

Human codon-optimized cDNA encoding the NA ectodomain (Genscript, USA) including the head and stalk of A/North Carolina/07/2013 (H1N1) (GenBank accession no. AGV29185.1; referred to as N1 NC13), A/Wisconsin/09/2013 (H1N1) (GenBank accession no. AGV29183.1; referred to as N1 WI13), A/Hunan/795/2002 (H5N1) (GenBank accession no. BAM85820.1; referred to as N1 HU02), A/Hong Kong/1968 (H3N2) (GenBank accession no. ABQ97206.1; referred to as N2 HK68), A/Germany/7830/2018 (H3N2) (GenBank accession no. QBH71200.1; referred to as N2 GE18), A/Singapore/1/57 (H2N2) (GenBank accession no. AY209895.1; referred to as N2 SI57) were cloned into pFRT expression plasmids (Thermo Fisher Scientific) as previously described^33,39^. The NA ectodomain sequences of N1 NC13 and N2 HK68 were preceded by sequences encoding the signal sequence derived from Gaussia luciferase, the SpyTag, a Twin-Strep-tag for affinity purification (IBA GmbH), and the Tetrabrachion tetramerization domain (similarly as described previously^33,39^). To generate the NA proteins for use in ELISAs the non-NA encoding sequences downstream of the signal peptide sequence were replaced, to ensure that any detected signal resulted from reactivity against the NA ectodomain. In these plasmids encoding N1 WI13, N1 HU02, N2 HK68, N2 GE18, and N2 SI57 the NA ectodomain sequences were preceded by the Gaussia luciferase signal sequence, a Flag tag for affinity purification, and the GCN4 tetramerization domain.

### NA protein expression and purification

NA proteins were expressed in Freestyle 293-F cells (Gibco) that were maintained in Freestyle 293 Expression Medium (Gibco) at 37 °C with 8% CO_2_ with shaking at 130 rpm. Plasmids encoding the NA proteins were transfected into the cells using polyethyleneimine (PEI) in a 1:3 ratio (µg DNA:µg PEI). After 24 hours Peptone Primatone RL (Sigma-Aldrich) and valproic acid (Sigma) were added to final concentrations of 0.5% and 2,25 mM, respectively. Cells were then incubated for a further 3–4 days until viability fell below 80%. Cell supernatant was cleared by centrifugation (30 x *g* for 15 min) and for the expressions containing the Strep-tag the supernatant was incubated with Biolock (IBA) for 20 minutes. Then, StrepTactin Sepharose resin (IBA) was added to the expressions containing the Strep-tag, or Pierce Anti-DYKDDDDK affinity resin (Thermo Fisher Scientific) to the expressions containing the Flag tag. After overnight incubation at 4 °C the proteins were purified on Poly-Prep chromatography columns (BioRad) according to instructions of the manufacturers of the affinity resins. Strep-tagged proteins were eluted using D(+)-Biotin (Roth) and Flag-tagged proteins using Pierce 3xDYKDDDDK peptide (Thermo Scientific). Proteins were analyzed for correct size and purity by sodium dodecyl sulfate polyacrylamide gel electrophoresis (SDS-PAGE) on a 12% gel and under reducing conditions, stained with GelCode Blue Stain Reagent (Thermo Scientific).

### Cryo-electron microscopy of recombinant NA proteins

Purified NA N1 was diluted 10x in PBS to a concentration of 0.15 mg/ml. 3 µL of the sample was loaded onto a Quantifoil 300 mesh 1.2/1.3 grid (Quantifoil Micro Tools) that was glow discharged (PELCO easiGlow, Ted Pella). The drop was blotted, and the sample was vitrified by plunging into a liquid ethane/propane mixture (37% ethane) using a Vitrobot Mark IV plunger (Thermo Fisher Scientific) set to 4 °C, 95% humidity, blot force 0, and blotting time 3 s.

Data was collected on a Talos Arctica (Thermo Fisher Scientific) transmission electron microscope operated at 200 kV, equipped with a postcolumn energy filter (Gatan) operated in zero-loss imaging mode with a 20-eV energy selecting slit. Micrographs were collected using a K2 Summit direct electron detector (Gatan) in counting mode, at a magnification of 130,000x (1.041 Å/pix). Exposure time for each micrograph was 8 s with 0.2 s per subframe, with a total dose of 48.1 e-/Å and a dose per subframe of 1.2 e^−^/Å. 1466 micrographs were recorded with a defocus range of −1 to −3 micrometers.

Motion and CTF correction were done in cryoSPARC^58^. A subset of particles was picked by the blob picker and was used to create templates for automated particle picking. Three rounds of template matching and reference-free 2D classification were performed, resulting in 199,724 particles. For the 3D refinement, a homogeneous refinement was applied. The map was segmented in ChimeraX^59^, and only the ‘head’ was imported into cryoSPARC to create a mask by extending the map by 3 pixels and adding a 15-pixel soft cosine edge. Applying a C4 symmetry with this mask, and subsequent CTF refinement and sharpening with a B-factor of −100 resulted in a 2.99 Å map, based on the “gold standard” FSC = 0.143 criterion (Figure S2c).

The initial model was built using ModelAngelo^60^ with the cryo-EM map and part of the sequence (residues 152–530). Additional residues were built (144–151) in the map and real-space refined using COOT^61^. Three iterations of real-space refinement manually in COOT and globally in PHENIX^62^ were performed.

### Mi3 nanoparticle expression, purification, and NA-Mi3 assembly

For the expression of the SpyCatcher-Mi3 nanoparticles^32^ the pET28a-SpyCatcher-Mi3 (Addgene plasmid #112255) plasmid was transformed into BL21 cells (Novagen). The cells were grown at 37 °C in LB medium until they reached log phase (OD_600_ ~ 0.8) and then expression was induced by adding isopropyl-β-d-thiogalactopyranoside (IPTG; GIBCO BRL) to a final concentration of 0.5 mM. Cultures were incubated for 16 hours at 22 °C, then pelleted and resuspended in lysis buffer (50 mM HEPES, 150 mM NaCl, 0.1% Triton X-100, 0.1 mg/mL Lysozyme, cOmplete Protease Inhibitor (Roche)). Samples were then sonicated on ice in four rounds of 30 seconds. Debris was removed by ultracentrifugation (76,000 x *g* for 45 min at 5 °C) and supernatant was incubated overnight with CaptureSelect C-Tag affinity matrix (Thermo Scientific). Nanoparticles were purified according to manufacturer’s instructions and eluted with 2 M MgCl_2_. Proteins were analyzed on 12% SDS-PAGE gel with GelCode Blue (Thermo Scientific). NA-Mi3 nanoparticles were assembled by co-incubation of SpyTag-NA and SpyCatcher-Mi3 in 50 mM HEPES and 150 mM NaCl pH 7.4 For 16 hours at room temperature. Conjugation of NA to the nanoparticles was confirmed with SDS-PAGE on a 12% gel under reducing conditions, stained with GelCode Blue (Thermo Scientific). Gels shown per figure were derived from the same experiment and were processed in parallel.

### Negative stain electron microscopy of NA-Mi3 nanoparticles

Mi3 nanoparticles or Mi3-N1 nanoparticles were diluted to 0.1 mg/ml in 50 mM HEPES and 150 mM NaCl pH 7.4, on ice. Subsequently, 3 μl of the sample was applied to carbon-coated copper grids that had been glow discharged for 30 seconds using a Cressington 208 instrument. The sample was allowed to absorb for 30 seconds prior to 2X wash with MQ and staining with 2% uranyl acetate solution. Grids were allowed to dry in air for ∼5 min and then imaged on a FEI Tecnai 20 transmission EM operated at 200 keV, equipped with a CCD camera.

### Cryo-electron tomography of NA-Mi3 nanoparticles

Both uncoupled and coupled nanoparticles were used at a concentration of 0.2 mg/ml. 4 µL of sample was loaded onto a Quantifoil 2/1 grid (Quantifoil Micro Tools) that was glow discharged (PELCO easiGlow, Ted Pella). 1 µL of a BSA-conjugated gold beads (Aurion) suspension was added and the drop was blotted from the back (other side of sample deposition) for 4–6 seconds. Sample was vitrified by plunge freezing in liquid ethane-propane mix (37% ethane) using a manual plunge-freezer (MPI-Martinsried).

Data was collected on a Talos Arctica (Thermo Fisher Scientific) transmission electron microscope operated at 200 kV, equipped with a postcolumn energy filter (Gatan) operated in zero-loss imaging mode with a 20-eV energy selecting slit. Tomograms were collected using a K2 Summit direct electron detector (Gatan) in counting mode with dose fractionation, at a magnification of 100,000x (1.359 Å/pix). SerialEM^63^ was used to record tilt series with a grouped dose-symmetric scheme, with a range from -45° to 45° in 3° increments. Target defocus was set at −4 micrometers and total dose approximately 110 e^-^/Å.

For tomogram reconstruction, the frames were aligned on-the-fly using Warp^64^ and reconstructed using IMOD^65^, using weighted back-projection and a SIRT-like filter^66^.

### MUNANA NA activity assay

To measure the activity of the purified NA proteins and determine whether conjugation to the nanoparticles impacted NA activity we performed a MUNANA assay on the unconjugated NA and NA -Mi3. This assay works on the principle that NA hydrolyzes the substrate 2′-(4-methylumbelliferyl)-α-d-N-acetylneuraminic acid (MUNANA; Sigma-Aldrich) to the fluorescent 4-methylumbelliferone (4-MU)^67^. The protein preparations were serially diluted in (50 mM Tris-HCl, 4 mM CaCl_2_, pH 6.0) in a flat-bottom 96-well black plate (Greiner Bio-One). An equal volume of reaction buffer containing 200 µM MUNANA was added to each well and the plate was incubated at 37 °C for one hour. The reaction was terminated by adding the stop solution (0.1 M glycine, 25% ethanol, pH 10.7). Fluorescence was measured immediately after stopping the reaction using Promega GloMax Explorer with excitation filter 365 nm and emission filter 415–445 nm.

### Mouse immunization experiments

All animal experiments were conducted according to the Belgian legislation (Belgian Law 14/08/1986 and Belgium Royal Decree 06/04/2010) and European legislation on the protection of animals used for scientific purposes (EU directives 2010/63/EU and 86/609/EEC). Experimental protocols were all approved by the Ethics Committee of the Vlaams Instituut voor Biotechnologie (VIB), Ghent University, Faculty of Science (permit numbers EC2021–032 and EC2022–104). Six- to 8-week-old female BALB/c mice (Charles River) were housed under specific-pathogen-free conditions with food and water *ad libitum*. Animals were immunized subcutaneously in the left and right flank with 100 µl containing 1 µg or 0.1 µg of recombinant protein (N1 or N2) or nanoparticle (N1-Mi3, N2-Mi3, or a combined formulation). All immunizations were adjuvanted with a 1:1 volume of Sigma Adjuvant System or SAS (S6322–1VL, Sigma). Mice received two immunizations in total (prime on D0 and boost on D21). Blood was collected on D21 & D42 by puncturing the lateral tail vein with a 23 G needle. In immunization experiments that were conducted for serology analysis and were not followed by viral challenge, mice were humanely sacrificed on D42 with an overdose of pentobarbital (Kela, Nembutal) and blood was collected by retro-orbital bleeding. The obtained blood samples were incubated overnight at 4 °C to allow clotting, which was followed by centrifugation at 6000 × *g* for 10 min. The supernatant (the serum) was recovered and submitted to a second centrifugation at the same speed. Cleared sera were stored at −20 °C before use in serological assays. On D42, mice were challenged with a 4LD50 dose of mouse-adapted A/Belgium/1/2009 [H1N1] (referred to as Bel/09) or X31 virus (A/Aichi/2/68 [H3N2] X PR8)^68,69^. Influenza virus infections were performed under sedation with a mixture of ketamine (Eurovet, Netamik, 10 mg/kg) and xylazine (Bayer, Rompun, 60 mg/kg) administered intraperitoneally, and a total of 50 μl of the virus inoculum was instilled equally across the nostrils of the mouse. After infection, the body weight of the mice was determined daily for two weeks. Animals that had lost ≥25% of their original body weight were humanely euthanized by cervical dislocation.

### Enzyme-linked immunosorbent assay

Antibody titers in mouse serum samples were determined using Enzyme-Linked Immunosorbent Assay (ELISA). NUNC MaxiSorp plates (Thermo Scientific) were coated with recombinant NA proteins containing the GCN4 tetramerization domain and Flag affinity tag diluted in Dulbecco’s PBS with Ca and Mg (Capricorn) and incubated overnight at 4 °C. The tetramerization domain and affinity tag were intentionally changed from the antigens used for immunizations to ensure that any signal detected in ELISA reflected antibody binding to the NA ectodomain specifically. The plates were washed three times with PBS + 0.05% Tween-20 and blocked for one hour with 3% bovine serum albumin and 0,1% Tween-20 in PBS. Serum samples were added to the plates in three-fold serial dilutions in a blocking buffer and incubated for two hours at room temperature. Starting dilutions were 1:100, 1:150, or 1:300, depending on the volume of the serum sample that was available. After incubation, the plates were washed four times and then incubated with Polyclonal Rabbit anti-Mouse Immunoglobulins-HRP (Dako P0260; 1:1000) in blocking buffer for one hour. Plates were then washed three times and incubated with TMB (BioFX) for 3–5 minutes before stopping the reaction with H_2_SO_4_. Read-out was performed using BioSPX 800 TS Microplate reader (BioTek) at 450 nm. Endpoint titers were determined as the reciprocal of the highest dilution with a signal above the background value. The background was determined as mean + 3*SD of the signal at the lowest dilution of control sera (either from mice immunized with PBS or unconjugated Mi3 nanoparticles).

### Viruses

Mouse-adapted A/Belgium/1/2009 (H1N1; referred to as Bel/09), A/Aichi/2/68 (H3N2 in the genetic background of PR8; referred to as X31) or A/Hong Kong/1/68 (H3N2 in the genetic background of PR8^69^; referred to as HK68) virus strains were amplified on Madin-Darby canine kidney (MDCK) cells in serum-free Dulbecco’s Modified Eagle medium (DMEM) supplemented with non-essential amino acids, 2 mM L-glutamine and 0.4 mM sodium pyruvate in the presence of 2 μg/mL TPCK-treated trypsin (T1426–50MG, Sigma) at 37 °C in 5% CO_2_. Ninety six hours after virus inoculation, the culture medium was collected, cell debris was removed by centrifugation for 10 min at 2500 x *g* at 4 °C, and the virus was pelleted from the supernatants by overnight centrifugation at 30,000 x *g* at 4 °C. The pellet was resuspended in cold sterile 20% glycerol in PBS, aliquoted, and stored at −80 °C until used.

#### Enzyme-linked Lectin assay

Neuraminidase inhibition titers were determined with an Enzyme-Linked Lectin Assay (ELLA). This assay measures the ability of the serum samples to inhibit sialic acid cleavage from the glycoprotein fetuin by H1N1 or H3N2 IAV. Fifty µl of a 25 µg/ml fetuin solution (Sigma cat. # F3385; dissolved in PBS) was coated in wells of Nunc MaxiSorp™ plates (ThermoFisher cat. # 44–2404–21), which were incubated overnight at 4 °C. The plates were then washed three times with PBS containing 0.05% Tween-20 (PBS-T). Serum samples were heat inactivated by incubation at 56 °C for 1 h and a 2-fold serial dilution series was made in sample buffer (1X MES VWR cat. # AAJ61979-AP: 20 mM CaCl_2_, 1% BSA, 0.5% Tween-20). Sixty µl of the diluted sera was pre-mixed with 60 µl of a 1/1250 dilution of a 3.3x10^8^ PFU/ml stock of A/Belgium/1/2009 (Bel/09) or a 1/103658,5 dilution of a 1.7 x 10^7^ PFU/ml stock of A/Hong Kong/1/68 (HK68) virus. These virus dilutions correspond to the 70% maximum activity of NA from the respective viruses as determined in the ELLA assay. Fifty µl of the serum:virus mixture was added to the fetuin plates in duplicate and the plates were further incubated at 37 °C for 16–18 h. The plates were then washed six times with PBS-T and incubated for 1 h with a solution of PNA-HRP (cat. # L6135–1MG, Sigma) at 5 μg/ml in conjugate diluent (MES pH 6.5, 20 mM CaCl_2_, 1% BSA). After three washes with PBS-T, TMB substrate (cat # 555214, BD Pharmingen) was added, and the plates were incubated for 5 min before the reaction was stopped by the addition of 1 M H_2_SO_4_. The optical density was measured at 450 nm and as a reference 655 nm in a Tecan microplate reader M200. Half maximum inhibitory concentrations (IC_50_) values were determined by nonlinear regression analysis (GraphPad Prism software).

### Statistical analysis ELISA and ELLA

Data were analyzed using GraphPad Prism version 9.3.1. Horizontal bars represent mean values and data plotted with error bars represent means with s.d. Statistical differences between groups were analyzed with Kruskal-Wallis and Dunn’s post-hoc test on log-transformed titers. The differences between prime and boost titers (Supplementary Fig. 4) were analyzed with the Mann-Whitney U test.

### Statistical analysis of virus challenge

Mouse body weights were recorded daily during the 14-day course of the challenge experiments with Bel/09 and X31 (experiments shown in Fig. 4). The data were analyzed in R statistical software^70^ using tidyverse^71^. We used a piecewise linear mixed model with knots at days 4 and 6 for both settings (Bel/09 and X31). Knots were chosen after careful exploratory data analysis at time points that capture inflection in the weight curves best over the different challenge experiments. The models were saturated in the fixed effects (treatment and all time segments) thus allowing for all possible interactions (up to 2-way). We also used a random intercept per mouse to model within-mouse correlation over time. Fitting was done using the lme4 package^72^ with the nlminb algorithm from the optimx package^73^. We then used robust covariance estimators from the clubSandwich package^74^ (vcovCR, type ‘CR0’) in conjunction with the multcomp package^75^ to calculate adjusted *p*-values and/or adjusted confidence intervals for contrasts pertaining to the research questions (Supplementary Tables 1–4).

For the challenge experiments comparing the homotypic, mixed, and mosaic nanoparticle vaccines the results of two independent experiments were analyzed together. Mouse body weights were recorded daily during the 14-day course of the challenge experiments with Bel/09 and X31 (experiments shown in Fig. 6). The data were analyzed in R statistical software^70^, using tidyverse^71^. Before modeling the data in Fig. 6, we selected only the groups of interest (hybrid, mixed, and N1 particles in the Bel09 setting and hybrid, mixed, and N2 particles in the X31 setting). We used the same type of model for all settings: a piecewise linear mixed model with knots at days 4 and 6. The models were saturated in the fixed effects (treatment, experiment, and all time segments) thus allowing for all possible interactions (up to 3-way). We used a random intercept and slope (for time) per mouse to model within-mouse correlation over time. Fitting was done using the lme4 package^72^ with the nlminb algorithm from the optimx package^73^. We then used robust covariance estimators from the clubSandwich package^74^ (vcovCR, type ‘CR0’) in conjunction with the multcomp package^75^ to calculate adjusted *p*-values and/or adjusted confidence intervals for contrasts pertaining to the research questions (Supplementary Tables 5 and 6).

### Reporting summary

Further information on research design is available in the Nature Research Reporting Summary linked to this article.

### Supplementary information

Supplemental Information

Reporting summary

## Data Availability

The cryo-EM structure shown in Fig. 1 is available via PDB ID: 9EWQ and EMDB ID: EMD-50022. All other data supporting the findings of this study are available within the paper and its Supplementary Information. Data underlying the results are shown in the different graphs in Figs. 2–6 and Supplementary Figs. 1 and 3–8 are available upon request via e-mail without any restrictions. NA expression constructs will be made available upon request by the authors under a material transfer agreement.
