# Supplementary material for: Nanoparticle display of neuraminidase elicits enhanced antibody responses and protection against influenza A virus challenge
Source: NPJ Vaccines. 2024 May 31;9:97. doi: 10.1038/s41541-024-00891-3 (PMC11143307; doi:10.1038/s41541-024-00891-3)
Supplement: Supplementary file 1 — Supplemental Information [file 41541_2024_891_MOESM1_ESM.pdf]

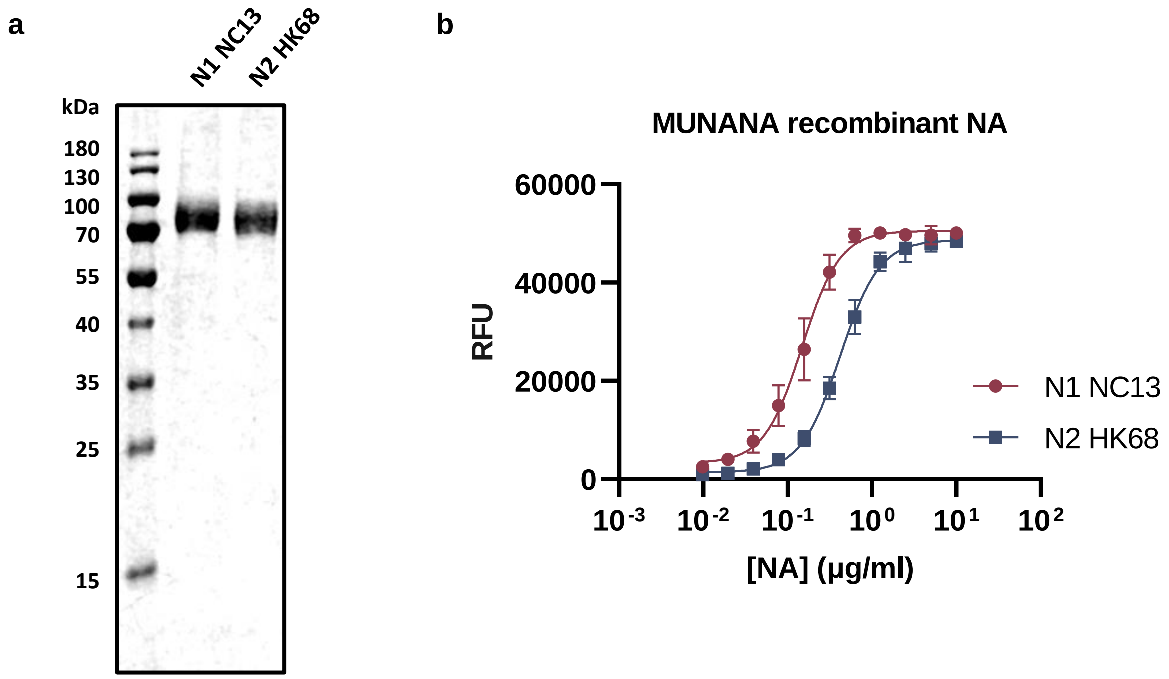

Supplementary figure 1. NA recombinant proteins quality control. (a) Representative image of purified recombinant soluble N1 NC13 and N2 HK68 proteins separated on Coomassie stained SDS-PAGE under reducing conditions. (b) Enzymatic activity of purified recombinant NA proteins determined in a MUNANA assay.

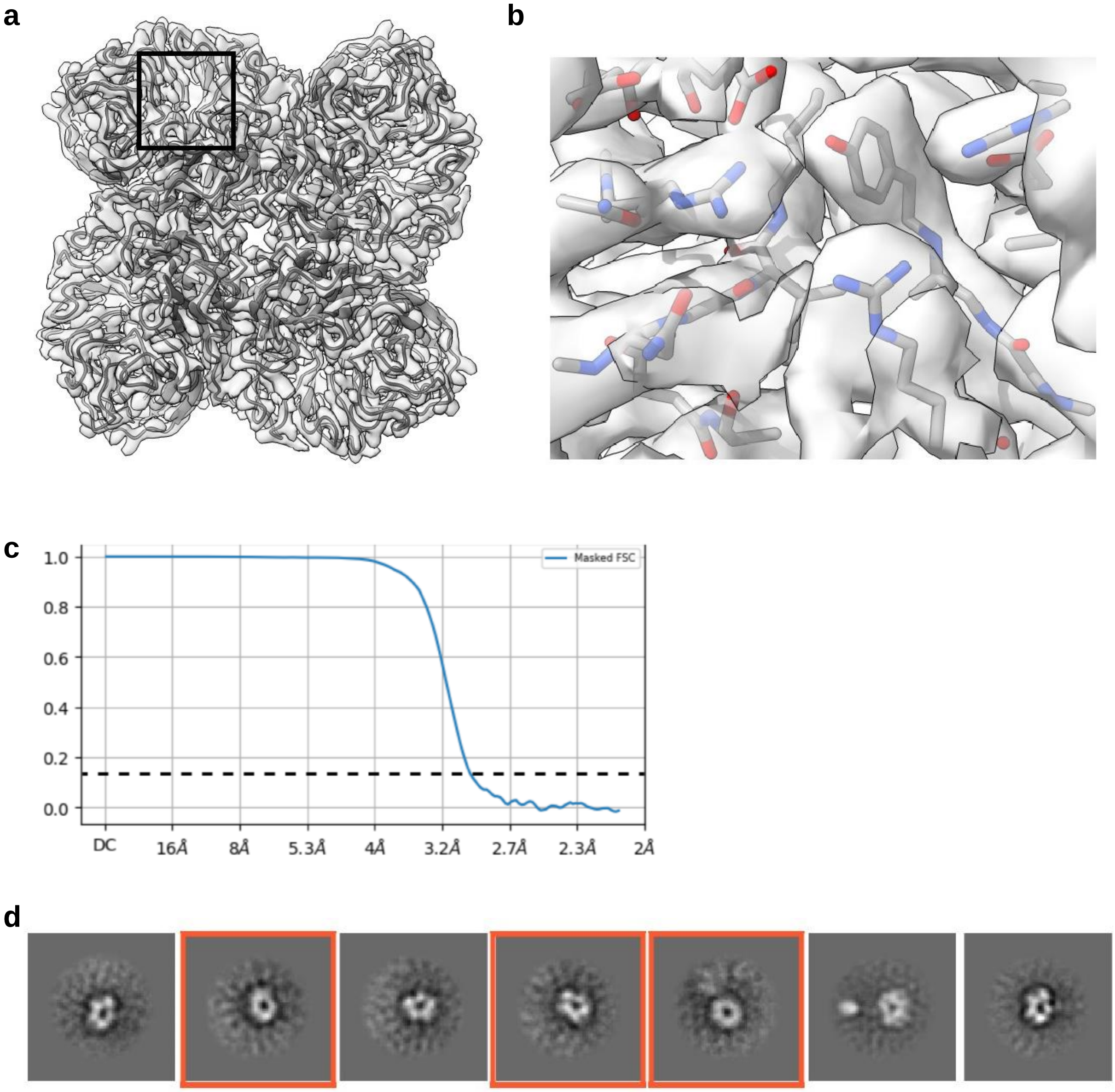

Supplementary figure 2. Structural characterization of NA antigens. (a) N1 NC13 cryo-EM map with the atomic model built. (b) Zoom in of the boxed region in (a) with a stick representation of the atomic model. (c) FSC curve of masked map after B-factor sharpening. The dotted line represents the cut-off at 0.143. (d) Negative stain of 2D classes of recombinant N2 HK68 stabilized with tetrabrachion domain display the closed conformation, top views marked in red.

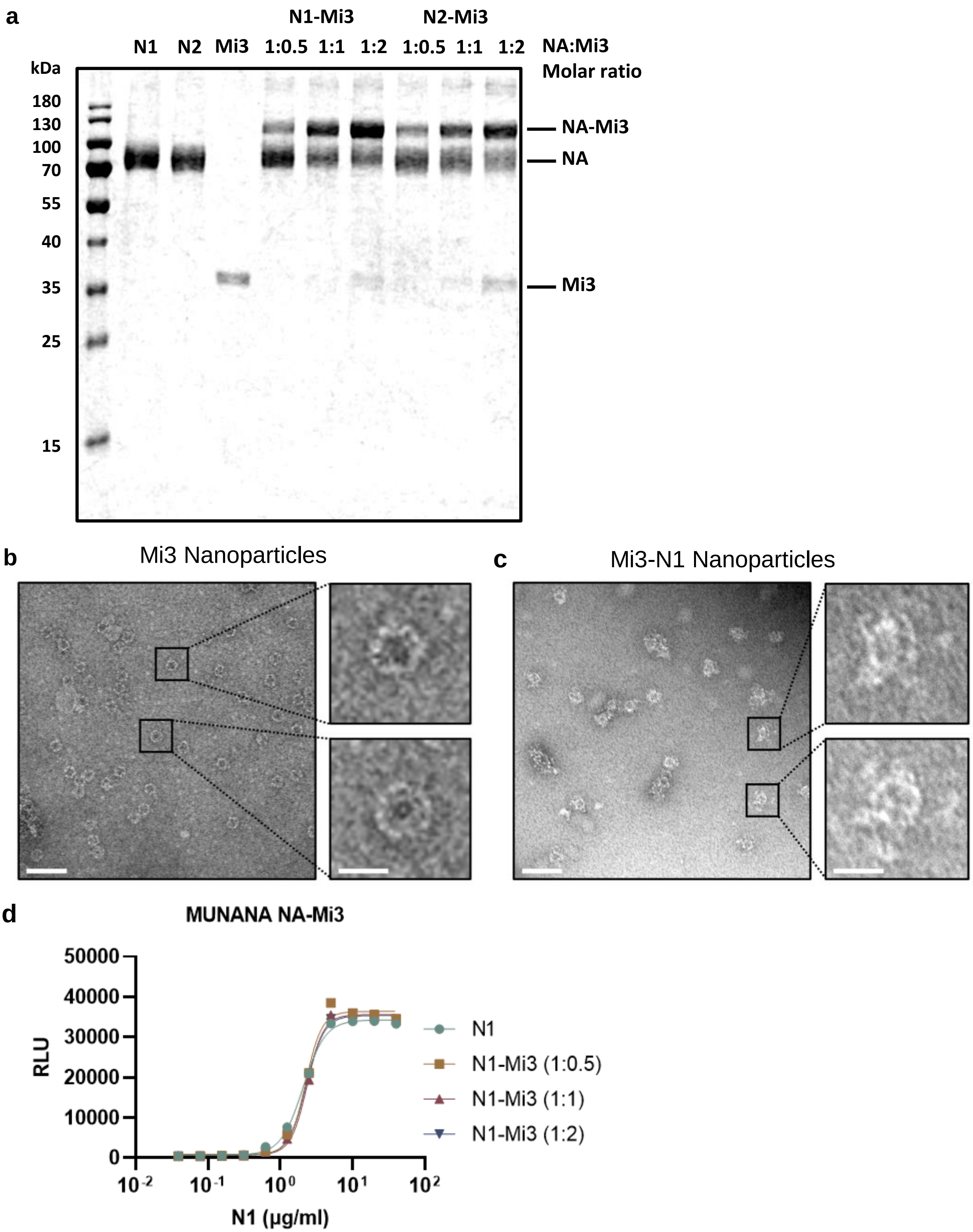

Supplementary figure 3. Conjugation of NA antigens to Mi3 nanoparticles. (a) Conjugation of NA-SpyTag to Mi3-SpyCatcher nanoparticles in varying ratios was demonstrated by reduced electrophoretic mobility on SDS-PAGE. Marker and first two lanes with NA proteins are the same as depicted in Fig. S1a. (b) Electron micrograph of negatively stained Mi3 nanoparticles. (c) Electron micrograph of negatively stained Mi3-N1 nanoparticles. Scale bars in main micrographs = 100 nm, scale bars in inset panels = 25 nm. (d) Enzymatic activity of unconjugated N1 and N1-Mi3 were compared at equal molar concentrations of N1 in the MUNANA assay.

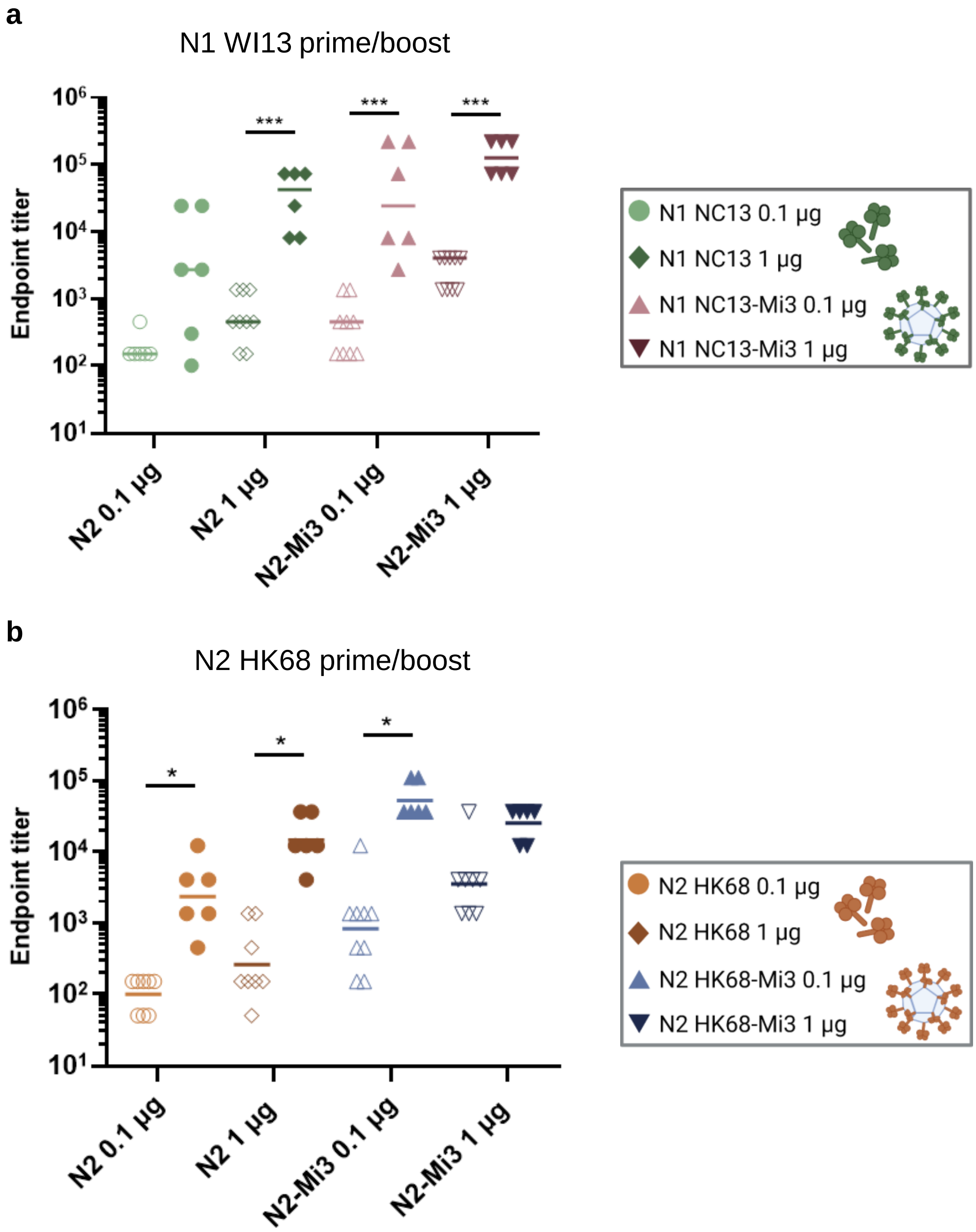

Supplementary figure 4. NA-binding endpoint titers prime vs. boost serum. Endpoint titers of antibodies binding N1 WI13 (a) from mice immunized with N1 NC13 and N1 NC13-Mi3 (Fig. 2b and c) and N2 HK68 (b) from mice immunized with N2 HK68 and N2 HK68-Mi3 (Fig. 3a and b). Data from sera taken after prime and boost immunizations are graphed to display the increase in endpoint titers. Open symbols: day 21 (post-prime). Closed symbols: day 42 (post-boost). Bars represent the geometric means for each group. Groups were compared with Mann-Whitney U test using GraphPad Prism 9.3.1 and statistical significance is indicated (*, $P < 0.05$; ***, $P < 0.001$).

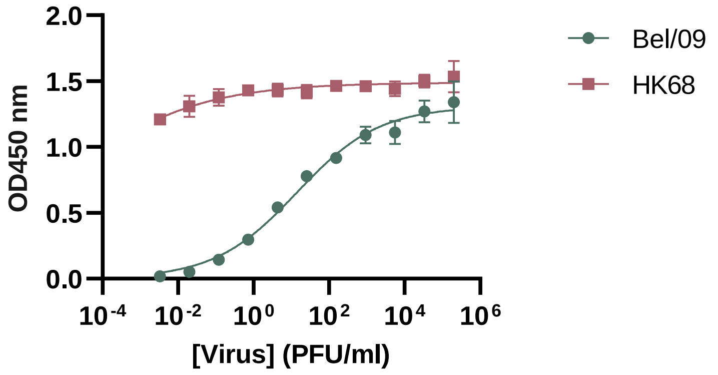

Supplementary figure 5. H3N2 HK68 has higher NA enzymatic activity than H1N1 Bel/09. NA activity of H3N2 HK68 and H1N1 Bel/09 quantified in an ELLA assay. A dilution series of the viruses was incubated in wells coated with the glycoprotein fetuin and the removal of sialic acids was detected by binding of PNA lectin (mean ±SD).

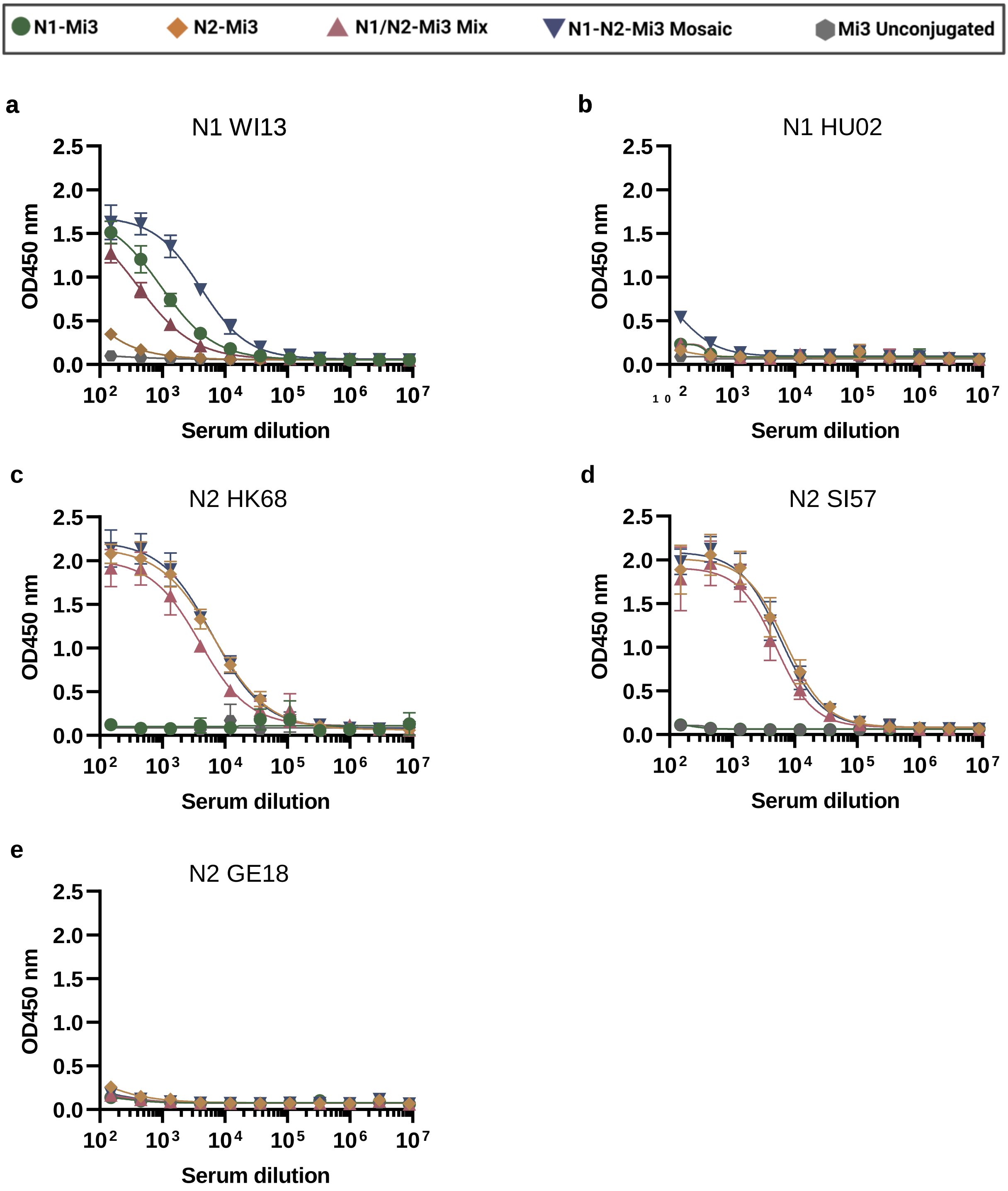

Supplementary figure 6. Cross-reactivity of serum from mice immunized with NA-Mi3 nanoparticles with heterologous NAs. Sera from mice (n=12) immunized with homotypic, mix, or mosaic NA-Mi3 formulations taken at day 42 post-immunization were pooled for the individual groups. NA-binding antibodies in the pooled serum samples were quantified in ELISA. (a and c) Reactivity against homologous N1 WI13 and N2 HK68. (b, d, and e) Reactivity against heterologous N1 (HU02) and N2 (SI57 and GE18) NAs. Symbols indicate the mean of three technical replicates and error bars indicate the standard deviation.

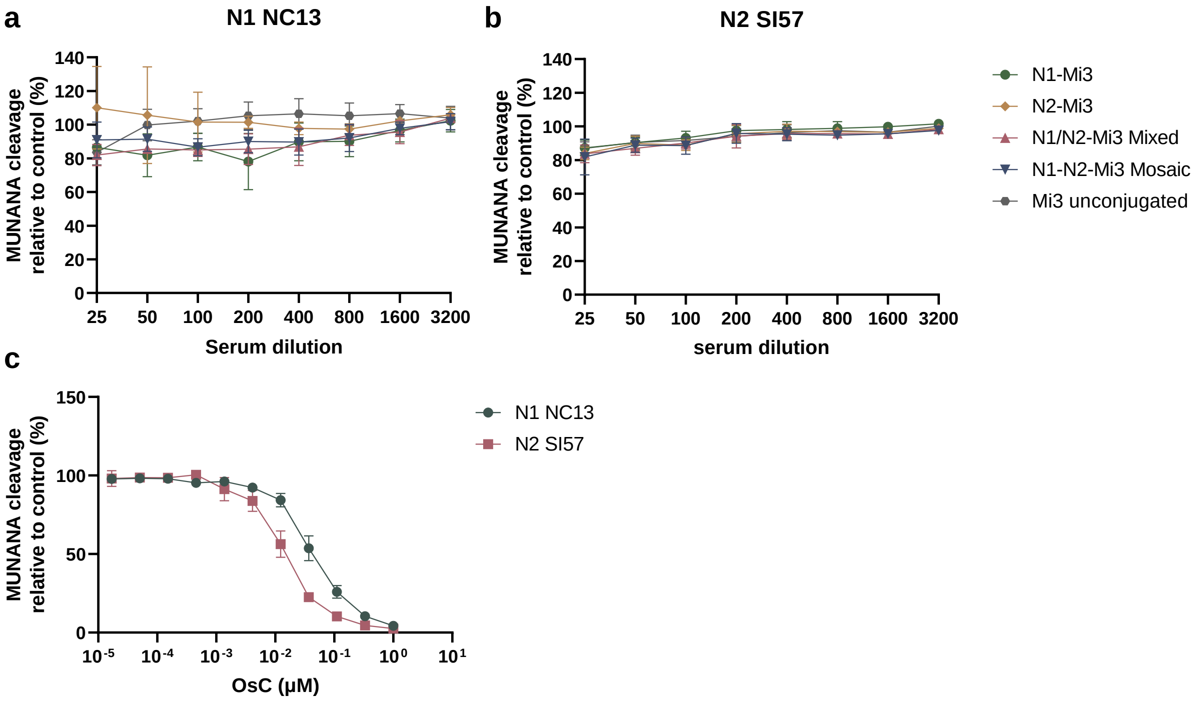

Supplementary figure 7. NA-Mi3 immunization does not induce antibodies capable of inhibiting NA activity in a small-molecule inhibition assay. Sera from mice (n=12) immunized with homotypic, mix, or mosaic NA-Mi3 formulations taken at day 42 post-immunization were pooled for the individual groups. NA inhibition assays were performed using the small-molecule substrate MUNANA to determine whether antibodies elicited by NA-Mi3 immunizations bind directly in the NA catalytic site. No inhibition of the MUNANA cleavage activity of N1 NC13 (a) or N2 SI57 (b) by any of the pooled sera was detected, while the small-molecule NA inhibitor oseltamivir carboxylate (OsC) inhibits cleavage activity of both NAs (c). Symbols indicate the mean of three (c) or four (a and b) technical replicates and error bars indicate the standard deviation.

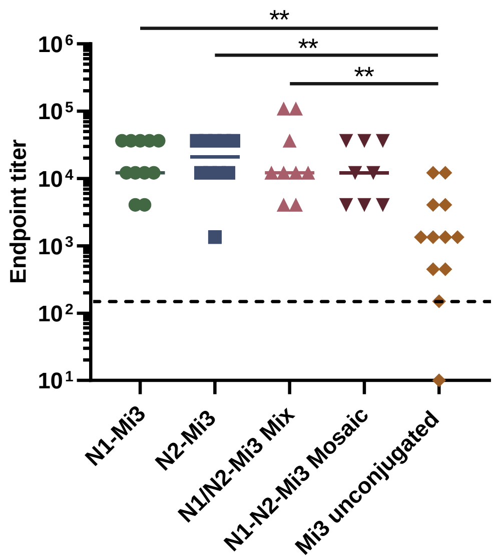

Supplementary figure 8. Anti-scaffold antibodies induced by (NA)-Mi3 nanoparticles. Antibodies binding the Mi3 nanoparticle carrier in sera from mice (n=12) immunized with homotypic NA-Mi3 or combined formulations taken at day 42 post-immunization were quantified in ELISA and endpoint titers were determined. Groups were compared with Kruskal-Wallis and Dunn's post-hoc test using GraphPad Prism 9.3.1 (**, $P < 0.01$).

Supplementary note 1

Body weight data depicted in Figures 4b,d and 6a,b were analyzed and modelled in R as described in Materials and Methods. Adjusted p-values and/or adjusted confidence intervals for contrasts pertaining to the research questions were calculated.

Supplementary Table 1. Model output for Figure 4b

	Effect[1]	Group	Term	Estimate	std.error	
1	fixed		Intercept	21,6	0,665	
2	fixed		Day	-0,903	0,0689	
3	fixed		Day4	0,428	0,174	
4	fixed		Day6	0,855	0,149	
5	fixed		Treatment 1 µg N1 NC13	1,18	0,94	
6	fixed		Treatment 0.1 µg N1 NC13-Mi3	-1,52	0,94	
7	fixed		Treatment 1 µg N1 NC13-Mi3	-1,04	0,94	
8	fixed		Day:Treatment 1 µg N1 NC13	0,0601	0,0974	
9	fixed		Day:Treatment 0.1 µg N1 NC13-Mi3	0,376	0,0974	
10	fixed		Day:Treatment 1 µg N1 NC13-Mi3	0,409	0,0974	
11	fixed		Day4:Treatment 1 µg N1 NC13	0,538	0,24	
12	fixed		Day4:Treatment 0.1 µg N1 NC13-Mi3	0,407	0,24	
13	fixed		Day4:Treatment 1 µg N1 NC13-Mi3	0,419	0,24	
14	fixed		Day6:Treatment 1 µg N1 NC13	-0,656	0,199	
15	fixed		Day6:Treatment 0.1 µg N1 NC13-Mi3	-1,03	0,199	
16	fixed		Day6:Treatment 1 µg N1 NC13-Mi3	-1,05	0,199	
17	random	ID	SD Intercept	1,57		
18		Residual	SD Observation	0,556		

[1] N1 NC13 vs N1 NC13-Mi3; H1N1 Bel/09 challenge

Supplementary table 2. Specific contrasts for experimental questions for Figure 4b

Contrast	Estimate	std.error	Estimate (% of 22g)	Adj. p-value	95% CI, lower	95% CI, upper
Weight change rate N1 NC13 0.1 µg until Day 4	-0.90	0.15	-4.1	<0.001	-1.32	-0.48
Weight change rate N1 NC13 1 µg until Day 4	-0.84	0.08	-3.8	<0.001	-1.08	-0.61
Weight change rate N1 NC13-Mi3 0.1 µg until Day 4	-0.53	0.07	-2.4	<0.001	-0.72	-0.33
Weight change rate N1 NC13-Mi3 1 µg until Day 4	-0.49	0.10	-2.2	<0.001	-0.77	-0.22
1 vs 0.1 µg N1 NC13, difference in weight change rate until Day 4	0.06	0.17	0.3	1	-0.42	0.54
1 vs 0.1 µg N1 NC13-Mi3, difference in weight change rate until Day 4	0.03	0.12	0.2	1	-0.31	0.37
N1 NC13-Mi3 vs N1 NC13, difference in weight change rate until Day 4, averaged over concentration	0.36	0.10	1.6	0.006	0.07	0.66
Weight change rate 0.1 µg N1 NC13 Day 4 - 6	-0.48	0.24	-2.2	0.34	-1.14	0.19
Weight change rate 1 µg N1 NC13 Day 4 - 6	0.12	0.13	0.6	0.957	-0.23	0.48
Weight change rate 0.1 µg N1 NC13-Mi3 Day 4 - 6	0.31	0.06	1.4	<0.001	0.15	0.47
Weight change rate 1 µg N1 NC13-Mi3 Day 4 - 6	0.35	0.11	1.6	0.013	0.05	0.66
1 vs 0.1 µg N1 NC13, difference in weight change rate Day 4 - 6	0.60	0.27	2.7	0.22	-0.16	1.36

1 vs 0.1 µg N1 NC13-Mi3, difference in weight change rate Day 4 - 6	0.05	0.12	0.2	1	-0.30	0.39
N1 NC13-Mi3 vs N1 NC13, difference in weight change rate Day 4 - 6, averaged over concentration	0.51	0.15	2.3	0.007	0.09	0.92

Supplementary Table 3. Model output for Figure 4d

	**Effect[1]**	**Group**	**Term**	**Estimate**	**std.error**	
1	fixed		Intercept	23,2	0,425	
2	fixed		Day	-1,28	0,0636	
3	fixed		Day4	1,37	0,16	
4	fixed		Day6	0,254	0,134	
5	fixed		Treatment 1 µg N2 HK68	-0,438	0,601	
6	fixed		Treatment 0.1 µg N2 HK68-Mi3	-2,49	0,601	
7	fixed		Treatment 1 µg N2 HK68-Mi3	1,57	0,601	
8	fixed		Day:Treatment 1 µg N2 HK68	0,466	0,0899	
9	fixed		Day:Treatment 0.1 µg N2 HK68-Mi3	0,435	0,0899	
10	fixed		Day:Treatment 1 µg N2 HK68-Mi3	1,2	0,0899	
11	fixed		Day4:Treatment 1 µg N2 HK68	0,0656	0,221	
12	fixed		Day4:Treatment 0.1 µg N2 HK68-Mi3	0,118	0,221	
13	fixed		Day4:Treatment 1 µg N2 HK68-Mi3	-1,44	0,221	
14	fixed		Day6:Treatment 1 µg N2 HK68	-0,768	0,18	
15	fixed		Day6:Treatment 0.1 µg N2 HK68-Mi3	-0,808	0,18	
16	fixed		Day6:Treatment 1 µg N2 HK68-Mi3	-0,0568	0,18	
17	random	ID	SD Intercept	0,964		
18		Residual	SD Observation	0,513		

[1] N2 HK68 vs N2 NCHK68-Mi3; H3N2 X31 challenge

Supplementary Table 4. Specific contrasts for experimental questions for Figure 4d

**Contrast**	**Estimate**	**std.error**	**Estimate (% of 22g)**	**Adj. p-value**	**95% CI, lower**	**95% CI, upper**
Weight change rate 0.1 µg protein N2 HK68 until Day 4	-1.28	0.05	-5.8	<0.001	-1.42	-1.13
Weight change rate 1 µg protein N2 HK68 until Day 4	-0.81	0.07	-3.7	<0.001	-1.00	-0.62
Weight change rate 0.1 µg N2 HK68-Mi3 until Day 4	-0.84	0.11	-3.8	<0.001	-1.15	-0.53
Weight change rate 1 µg N2 HK68-Mi3 until Day 4	0.07	0.04	0.3	0.39	-0.03	0.18
1 vs 0.1 µg N2 HK68, difference in weight change rate until Day 4	0.47	0.09	2.1	<0.001	0.23	0.71
1 vs 0.1 µg N2 HK68-Mi3, difference in weight change rate until Day 4	0.77	0.12	3.5	<0.001	0.44	1.10
N2 HK68-Mi3 vs N2 HK68, difference in weight change rate until Day 4, averaged over concentration	0.59	0.07	2.7	<0.001	0.38	0.79
Weight change rate 0.1 µg N2 HK68 Day 4 - 6	0.09	0.31	0.4	1	-0.78	0.96
Weight change rate 1 µg N2 HK68 Day 4 - 6	0.62	0.03	2.8	<0.001	0.52	0.72
Weight change rate 0.1 µg N2 HK68-Mi3 Day 4 - 6	0.64	0.10	2.9	<0.001	0.36	0.92
Weight change rate 1 µg N2 HK68-Mi3 Day 4 - 6	0.15	0.07	0.7	0.221	-0.04	0.33
1 vs 0.1 µg N2 HK68, difference in weight change rate Day 4 - 6	0.53	0.32	2.4	0.552	-0.35	1.41

Supplementary Table 5. Model output for Figure 6

	Effect[1]	Group	Term	Estimate	std.error	
**H1N1 Bel/09**						
1	fixed		Intercept	22	0,416	
2	fixed		Day	-0,295	0,0525	
3	fixed		Day4	0,498	0,122	
4	fixed		Day6	-0,182	0,0967	
5	fixed		N1/N2-Mi3 Mix	0,87	0,588	
6	fixed		N1-Mi3	-0,267	0,588	
7	fixed		Experiment2	-0,89	0,588	
8	fixed		Day:N1/N2-Mi3 Mix	-0,158	0,074	
9	fixed		Day:N1-Mi3	-0,371	0,074	
10	fixed		Day4:N1/N2-Mi3 Mix	0,121	0,180	
11	fixed		Day4:N1-Mi3	0,685	0,173	
12	fixed		Day6:N1/N2-Mi3 Mix	0,033	0,148	
13	fixed		Day6:N1-Mi3	-0,235	0,137	
14	fixed		Day:Experiment2	-0.029	0,074	
15	fixed		Day4:Experiment2	0,354	0,173	
16	fixed		Day6:Experiment2	-0,287	0,136	
17	fixed		N1/N2-Mi3 Mix:Experiment2	-0,532	0,831	
18	fixed		N1-Mi3:Experiment2	0,777	0,831	
19	fixed		Day: N1/N2-Mi3 Mix:Experiment2	0,025	0,105	
20	fixed		Day: N1-Mi3:Experiment2	0,073	0,105	
21	fixed		Day4: N1/N2-Mi3 Mix:Experiment2	-0,029	0,250	
22	fixed		Day4: N1-Mi3:Experiment2	-0,270	0,245	
23	fixed		Day6: N1/N2-Mi3 Mix:Experiment2	0,040	0,202	
24	fixed		Day6: N1-Mi3:Experiment2	0,219	0,193	
25	random	ID	SD Intercept	0.967		
26	random	ID	SD Day	0.028		
27	random	ID	Correlation Intercept/Day	-0.090		
28		Residual		0.414		
**H3N2 X31**						
1	fixed		Intercept	22,2	0,538	
2	fixed		Day	-0,507	0,073	
3	fixed		Day4	0,93	0,17	
4	fixed		Day6	-0,378	0,134	
5	fixed		N1/N2-Mi3 Mix	0,352	0,761	
6	fixed		N2-Mi3	-0,148	0,761	
7	fixed		Experiment2	-0,619	0,761	
8	fixed		Day:N1/N2-Mi3 Mix	-0,255	0,103	
9	fixed		Day:N2-Mi3	0,136	0,103	
10	fixed		Day4:N1/N2-Mi3 Mix	0,679	0,240	
11	fixed		Day4:N2-Mi3	-0,581	0,240	
12	fixed		Day6:N1/N2-Mi3 Mix	-0,396	0,190	
13	fixed		Day6:N2-Mi3	0,511	0,190	
14	fixed		Day:Experiment2	0,026	0,103	

15	fixed		Day4:Experiment2	0,111	0,240	
16	fixed		Day6:Experiment2	-0,085	0,190	
17	fixed		N1/N2-Mi3 Mix:Experiment2	0,307	1,076	
18	fixed		N2-Mi3:Experiment2	0,233	1,076	
19	fixed		Day: N1/N2-Mi3 Mix:Experiment2	-0,038	0,146	
20	fixed		Day: N2-Mi3:Experiment2	0,099	0,146	
21	fixed		Day4: N1/N2-Mi3 Mix:Experiment2	-0,467	0,340	
22	fixed		Day4: N2-Mi3:Experiment2	0,220	0,340	
23	fixed		Day6: N1/N2-Mi3 Mix:Experiment2	0,572	0,268	
24	fixed		Day6: N2-Mi3:Experiment2	-0,379	0,268	
25	random	ID	SD Intercept	1,242		
26	random	ID	SD Day	0,041		
27	random	ID	Correlation Intercept/Day	-0,086		
28		Residual		0,574		

[1] N1/N2-Mi3 mix & N1-N2-Mi3 Mosaic; H1N1 Bel09 &H3N2 X31 challenge

Supplementary Table 6. Specific contrasts for experimental questions for Figure 6

Contrast	Estimate	std.error	Estimate (% of 22g)	Adj. p-value	95% CI, lower	95% CI, upper
**H1N1 Bel/09**						
Weight loss rate N1-N2-Mi3 Mosaic	-0.31	0.04	-1.4	<0.001	-0.42	-0.20
Weight loss rate N1-Mi3	-0.64	0.05	-2.9	<0.001	-0.77	-0.51
Weight loss rate N1/N2-Mi3 Mix	-0.46	0.06	-2.1	<0.001	-0.63	-0.28
N1-N2-Mi3 Mosaic vs N1-Mi3, difference in weight loss rate	0.36	0.09	1.6	0.001	0.10	0.62
N1/N2-Mi3 Mix vs N1-Mi3, difference in weight loss rate	0.19	0.08	0.9	0.145	-0.03	0.41
N1-N2-Mi3 Mosaic vs N1/N2-Mi3 Mix, difference in weight loss rate	0.15	0.08	0.7	0.353	-0.06	0.36
**H3N2 X31**						
Weight loss rate N1-N2-Mi3 Mosaic	-0.49	0.13	-2.2	<0.001	-0.84	-0.15
Weight loss rate N2-Mi3	-0.31	0.07	-1.4	<0.001	-0.50	-0.12
Weight loss rate N1/N2-Mi3 Mix	-0.77	0.10	-3.5	<0.001	-1.04	-0.49
N1-N2-Mi3 Mosaic vs N2-Mi3, difference in weight loss rate	-0.12	0.18	-0.5	0.992	-0.62	0.39
N1/N2-Mi3 Mix vs N2-Mi3, difference in weight loss rate	-0.46	0.12	-2.1	0.002	-0.80	-0.12
N1-N2-Mi3 Mosaic vs N1/N2-Mi3 Mix, difference in weight loss rate	0.27	0.16	1.2	0.472	-0.17	0.72
