# Supplementary material for: Nanoparticle display of neuraminidase elicits enhanced antibody responses and protection against influenza A virus challenge
Source: NPJ Vaccines. 2024 May 31;9:97. doi: 10.1038/s41541-024-00891-3 (PMC11143307; doi:10.1038/s41541-024-00891-3)
Supplement: Supplementary file 2 — Reporting summary [file 41541_2024_891_MOESM2_ESM.pdf]

nature portfolio

Corresponding author(s): Dr Cornelis de Haan

Last updated by author(s): YYYY-MM-DD

Reporting Summary

Nature Portfolio wishes to improve the reproducibility of the work that we publish. This form provides structure for consistency and transparency in reporting. For further information on Nature Portfolio policies, see our Editorial Policies and the Editorial Policy Checklist.

Statistics

For all statistical analyses, confirm that the following items are present in the figure legend, table legend, main text, or Methods section.

n/a	Confirmed	
☐	☒	The exact sample size (*n*) for each experimental group/condition, given as a discrete number and unit of measurement
☐	☒	A statement on whether measurements were taken from distinct samples or whether the same sample was measured repeatedly
☐	☒	The statistical test(s) used AND whether they are one- or two-sided<br>*Only common tests should be described solely by name; describe more complex techniques in the Methods section.*
☒	☐	A description of all covariates tested
☐	☒	A description of any assumptions or corrections, such as tests of normality and adjustment for multiple comparisons
☐	☒	A full description of the statistical parameters including central tendency (e.g. means) or other basic estimates (e.g. regression coefficient) AND variation (e.g. standard deviation) or associated estimates of uncertainty (e.g. confidence intervals)
☒	☐	For null hypothesis testing, the test statistic (e.g. *F*, *t*, *r*) with confidence intervals, effect sizes, degrees of freedom and *P* value noted<br>*Give P values as exact values whenever suitable.*
☒	☐	For Bayesian analysis, information on the choice of priors and Markov chain Monte Carlo settings
☒	☐	For hierarchical and complex designs, identification of the appropriate level for tests and full reporting of outcomes
☒	☐	Estimates of effect sizes (e.g. Cohen's *d*, Pearson's *r*), indicating how they were calculated

Our web collection on statistics for biologists contains articles on many of the points above.

Software and code

Policy information about availability of computer code

Data collection	N.A.
Data analysis	Data were analyzed using GraphPad Prism version 9.3.1 and by R statistical software using tidyverse.

For manuscripts utilizing custom algorithms or software that are central to the research but not yet described in published literature, software must be made available to editors and reviewers. We strongly encourage code deposition in a community repository (e.g. GitHub). See the Nature Portfolio guidelines for submitting code & software for further information.

Data

Policy information about availability of data

All manuscripts must include a data availability statement. This statement should provide the following information, where applicable:

- Accession codes, unique identifiers, or web links for publicly available datasets
- A description of any restrictions on data availability
- For clinical datasets or third party data, please ensure that the statement adheres to our policy

All data underlying the results shown in the different graphs in Fig 2-6 and Fig S1, S3-S8 are available upon request via e-mail without any restrictions. The cryo-EM structure shown in Fig. 1 is available via PDB ID: 9EWQ (coordinates) and EMDB: EMD-50022 (map).

Research involving human participants, their data, or biological material

Policy information about studies with human participants or human data. See also policy information about sex, gender (identity/presentation), and sexual orientation and race, ethnicity and racism.

Reporting on sex and gender	N.A.
Reporting on race, ethnicity, or other socially relevant groupings	N.A.
Population characteristics	N.A.
Recruitment	N.A.
Ethics oversight	N.A.

Note that full information on the approval of the study protocol must also be provided in the manuscript.

Field-specific reporting

Please select the one below that is the best fit for your research. If you are not sure, read the appropriate sections before making your selection.

☒ Life sciences ☐ Behavioural & social sciences ☐ Ecological, evolutionary & environmental sciences

For a reference copy of the document with all sections, see nature.com/documents/nr-reporting-summary-flat.pdf

Life sciences study design

All studies must disclose on these points even when the disclosure is negative.

Sample size	In order to reduce the number of mice needed, we do not make use of more mice than strictly necessary to be able to test a hypothesis. Based on experience we anticipated that with 6 mice per group per experiment, we could generate data that are normally distributed (Kolmogorov-Smirnov and Shapiro test) and continuous (Shapiro test).
Data exclusions	No data were excluded from the analyses
Replication	Animal experiments shown in Fig.2, 3 and 4 were not replicated. Animal experiments shown in Fig. 5 and 6 were replicated in an independent experiment and combined data are presented in the figures.
Randomization	Mice were randomly allocated to the different experimental groups.
Blinding	Mice were randomly allocated to different vaccination groups. After vaccination and viral challenge, body weight was determined daily. Investigators were not blinded.

Reporting for specific materials, systems and methods

We require information from authors about some types of materials, experimental systems and methods used in many studies. Here, indicate whether each material, system or method listed is relevant to your study. If you are not sure if a list item applies to your research, read the appropriate section before selecting a response.

Materials & experimental systems

n/a	Involved in the study	
☐	☒	Antibodies
☐	☒	Eukaryotic cell lines
☒	☐	Palaeontology and archaeology
☐	☒	Animals and other organisms
☒	☐	Clinical data
☒	☐	Dual use research of concern
☒	☐	Plants

Methods

n/a	Involved in the study	
☒	☐	ChIP-seq
☒	☐	Flow cytometry
☒	☐	MRI-based neuroimaging

Antibodies

Antibodies used	Polyclonal Rabbit anti-Mouse Immunoglobulins-HRP (Dako, REF P0260, LOT 41326080)
Validation	No primary antibodies were used.

Eukaryotic cell lines

Policy information about cell lines and Sex and Gender in Research

Cell line source(s)	293F cells (Gibco), MDCK cells (CMB cell bank repository)
Authentication	293F Cells were directly purchased from Gibo, but not authenticated; MDCK cells authenticated.
Mycoplasma contamination	Cells tested negative for mycoplasm
Commonly misidentified lines (See ICLAC register)	N.A.

Animals and other research organisms

Policy information about studies involving animals; ARRIVE guidelines recommended for reporting animal research, and Sex and Gender in Research

Laboratory animals	Six- to 8-week-old female BALB/c mice (Charles River)
Wild animals	N.A.
Reporting on sex	No sex-based analysis was performed. Only female mice were used because they can be co-housed in groups, wheras co-housed male mice tend to fight with each other.
Field-collected samples	N.A.
Ethics oversight	All animal experiments were conducted according to the Belgian legislation (Belgian Law 14/08/1986 and Belgium Royal Decree 06/04/2010) and European legislation on protection of animals used for scientific purposes (EU directives 2010/63/EU and 86/609/EEC). Experimental protocols were all approved by the Ethics Committee of the Vlaams Instituut voor Biotechnologie (VIB), Ghent University, Faculty of Science (permit numbers EC2021-032 and EC2022-104).

Note that full information on the approval of the study protocol must also be provided in the manuscript.

Plants

Seed stocks	N.A.
Novel plant genotypes	N.A.
Authentication	N.A.
